# Chitosan stimulates root hair callose deposition, endomembrane dynamics, and inhibits root hair growth

**DOI:** 10.1111/pce.15111

**Published:** 2024-09-13

**Authors:** Matěj Drs, Pavel Krupař, Eliška Škrabálková, Samuel Haluška, Karel Müller, Andrea Potocká, Lucie Brejšková, Natalia Serrano, Aline Voxeur, Samantha Vernhettes, Jitka Ortmannová, George Caldarescu, Matyáš Fendrych, Martin Potocký, Viktor Žárský, Tamara Pečenková

**Affiliations:** ^1^ Institute of Experimental Botany of the Czech Academy of Sciences Prague 6 Czech Republic; ^2^ Department of Experimental Plant Biology, Faculty of Science Charles University Prague 2 Czech Republic; ^3^ Université Paris‐Saclay, INRAE, AgroParisTech, Institut Jean‐Pierre Bourgin (IJPB) Versailles France

**Keywords:** arabidopsis, cell wall, defence, gene expression, signalling

## Abstract

Although angiosperm plants generally react to immunity elicitors like chitin or chitosan by the cell wall callose deposition, this response in particular cell types, especially upon chitosan treatment, is not fully understood. Here we show that the growing root hairs (RHs) of Arabidopsis can respond to a mild (0.001%) chitosan treatment by the callose deposition and by a deceleration of the RH growth. We demonstrate that the glucan synthase‐like 5/PMR4 is vital for chitosan‐induced callose deposition but not for RH growth inhibition. Upon the higher chitosan concentration (0.01%) treatment, RHs do not deposit callose, while growth inhibition is prominent. To understand the molecular and cellular mechanisms underpinning the responses to two chitosan treatments, we analysed early Ca^2+^ and defence‐related signalling, gene expression, cell wall and RH cellular endomembrane modifications. Chitosan‐induced callose deposition is also present in the several other plant species, including functionally analogous and evolutionarily only distantly related RH‐like structures such as rhizoids of bryophytes. Our results point to the RH callose deposition as a conserved strategy of soil‐anchoring plant cells to cope with mild biotic stress. However, high chitosan concentration prominently disturbs RH intracellular dynamics, tip‐localised endomembrane compartments, growth and viability, precluding callose deposition.

## Summary statement

The root hairs (RHs) of Arabidopsis, when exposed to elicitor chitosan, deposit callose and change the growth rate. This is accompanied by altered signalling, gene expression, and is linked to altered pectin methylation state. Our research highlights the evolutionarily conserved role of RHs in environmental sensing.

## 1 INTRODUCTION

Root hairs (RHs) are unicellular plant structures crucial for several aspects of root function: mechanical anchorage of a plant in a substrate, the absorption of water and essential nutrients due to increased root surface, and in root‐microbe interactions. In the model plant *Arabidopsis thaliana*, RH cell fate is determined by a position‐dependent signalling cascade governed by specific transcription factors, leading to the RH initiation, bulging and cylindrical/polarised tip growth (Carol & Dolan, 2002; Dolan, 1996; Parker et al., 2000; Vissenberg et al., 2020). Several root hair‐defective (RHD) genes, F‐actin cytoskeletal meshwork and secretory pathway play a prominent role in this process (Baluška et al., 2000; Ichikawa et al., 2014). The tip growth is supported by constant formation of the new cell wall components, including cellulose, hemicellulose and pectins (Cavalier et al., 2008; Mendrinna & Persson, 2015; Park et al., 2011; Rounds & Bezanilla, 2013). Recently a mechanism involving formation of an RH‐tip complex of demethylated pectins with cell wall matrix proteins has been found to be crucial for providing structural organisation of periodic circumferential rings compaction providing anisometric RH growth (Schoenaers et al., 2024).

In aerial plant parts, when a pathogen attacks the plant cell, pathogen‐associated molecular patterns (PAMPs) triggered immunity (PTI) and potentially effector‐triggered immunity are activated (Jones and Dangl, 2006; Ngou et al., 2021). One of the earliest defence responses to a pathogen attack is the deposition of β−1,3 glucan polysaccharide callose by which the plant reinforces cell walls and blocks the pathogen entry (Jacobs et al., 2003). In Arabidopsis, among 12 callose synthase genes, only one of them, *GSL5* or *PMR4* (*POWDERY MILDEW RESISTANT 4*), is expressed upon pathogenic infection or elicitor treatment, such as fungal cell wall derived chitin and chitosan, but also endogenous damage‐associated molecular patterns (DAMPs) (Denoux et al., 2008; Gómez‐Gómez et al., 1999; Luna et al., 2011; Verma & Hong, 2001; Vogel & Somerville, 2000).

The roots are constantly exposed to a mixture of microorganisms, and plants need to prevent the over‐activation of immunity in roots, mainly by restricting defence responses to specific root zones and cell layers (Chuberre et al., 2018). The PTI in roots comprises the ROS (reactive oxygen species) production, patchy callose deposition and growth rate modifications (Badri et al., 2008; Chuberre et al., 2018; Millet et al., 2010; Rich‐Griffin et al., 2020; Zhang et al., 2022). Upon injury of RH, callose can be deposited locally in the RH cell wall as well (Galway et al., 2011). It has been recently shown that the defence‐related callose synthase PMR4 is responsible for the RH callose deposition upon phosphate starvation (Okada et al., 2024). The RH can react to both beneficial and pathogenic bacteria by the enhancement of its growth, while elicitor treatments do not provoke prominent RH growth changes, except for the DAMP PEP1 (Okada et al., 2021; Pečenková et al., 2017; Zamioudis et al., 2013).

Both RH tip growth and pathogen sensing are under the control of development‐ and defence‐related receptor kinases, and binding of their cognate ligands can lead to the ROS production and/or activation of Ca^2+^‐permeable channels, such as CYCLIC NUCLEOTIDE GATED CHANNELS (CNGCs) (Ladwig et al., 2015). The CNGCs produce transients with specific patterns, further transmitted to transcriptional reprogramming of defence‐ or symbiosis‐related genes (Yuan et al., 2017). Recent studies have reported that CNGC6, CNGC9 and CNGC14 are crucial for RH tip polar growth (Brost et al., 2019; Ladwig et al., 2015). A phosphorylation activation of mitogen‐activated protein kinase (MAPK) cascade is another convergence point of signalling pathways. The MAPK3/6 signalling cascade plays a role in the RH growth (Rentel et al., 2004), as well as in plant immunity (including chitin‐activated defence), together with the second MAPK4‐comprising cascade (rev. in Zhang and Zhang [2022]).

The simplest way to study defence responses is by applying a single PAMP or immunity elicitor such as chitin, polymer of β‐1,4‐linked N‐acetylglucosamine or its deacetylated derivate chitosan. The chitin recognition via Chitin Elicitor Receptor Kinase 1 (CERK1) and lysin motif receptor kinases 4 and 5 (LYK4 and 5) activates the production of chitinases by plants; to avoid this recognition and hydrolysis by chitinases, fungi secrete chitin de‐N‐acetylases to convert chitin to chitosan (Cai et al., 2011; Miya et al., 2007; Wan et al., 2012). Fully deacetylated polymer units can still activate Ca^2+^ signalling‐related subset of cellular responses, which in case of overactivation provokes cell death independent of the CERK1/ROS/MAPK pathway (Ye et al., 2020). Chitosan functions as a PAMP (Iriti et al., 2006), but also as an antimicrobial factor causing intracellular ROS burst with subsequent oxidation of pathogen cell membrane fatty acids and plasma membrane permeabilization (Lopez‐Moya et al., 2017). Interestingly, high doses of chitosan in the rhizosphere of Arabidopsis, tomato or barley significantly arrest root development, probably via interference with auxin and gibberellic acid regulation or via activation of jasmonic (JA) and salicylic acid (SA) related genes in roots (Lopez‐Moya et al., 2017; Suwanchaikasem et al., 2022).

In this report, we compare the elicitation of plant immunity by two different chitosan concentrations, and we bring evidence of the plant capability to adjust the RH rate growth, as well as to reinforce the RH cell wall by the callose deposition. The observed RH‐specific effects are dependent on the chitosan concentration, but not its molecular size, and are present also in other plant species. Further, on the subcellular level, we tested the responsiveness of RH endomembranes and cytoskeleton to chitosan treatments. To understand the impact of the chitosan treatments on overall plant growth and defence, we performed Ca^2+^ and MAPK signalling assays and RNAseq analysis. We conclude that the RH callose deposition is the consequence of the mild biotic stress affecting the cells with the intensive growth rate, while the stronger biotic stress provokes almost immediate and more prominent tip‐localised endomembrane changes and RH growth cessation. Our results thus reveal the unexpected aspects of the RH growth‐defence trade‐off, and set the basis for possible future modifications of the balanced resistance and fitness.

## 2 RESULTS

### 2.1 Chitosan treatment induces concentration‐dependent root growth changes and callose deposition also in RHs

**Figure 1:**
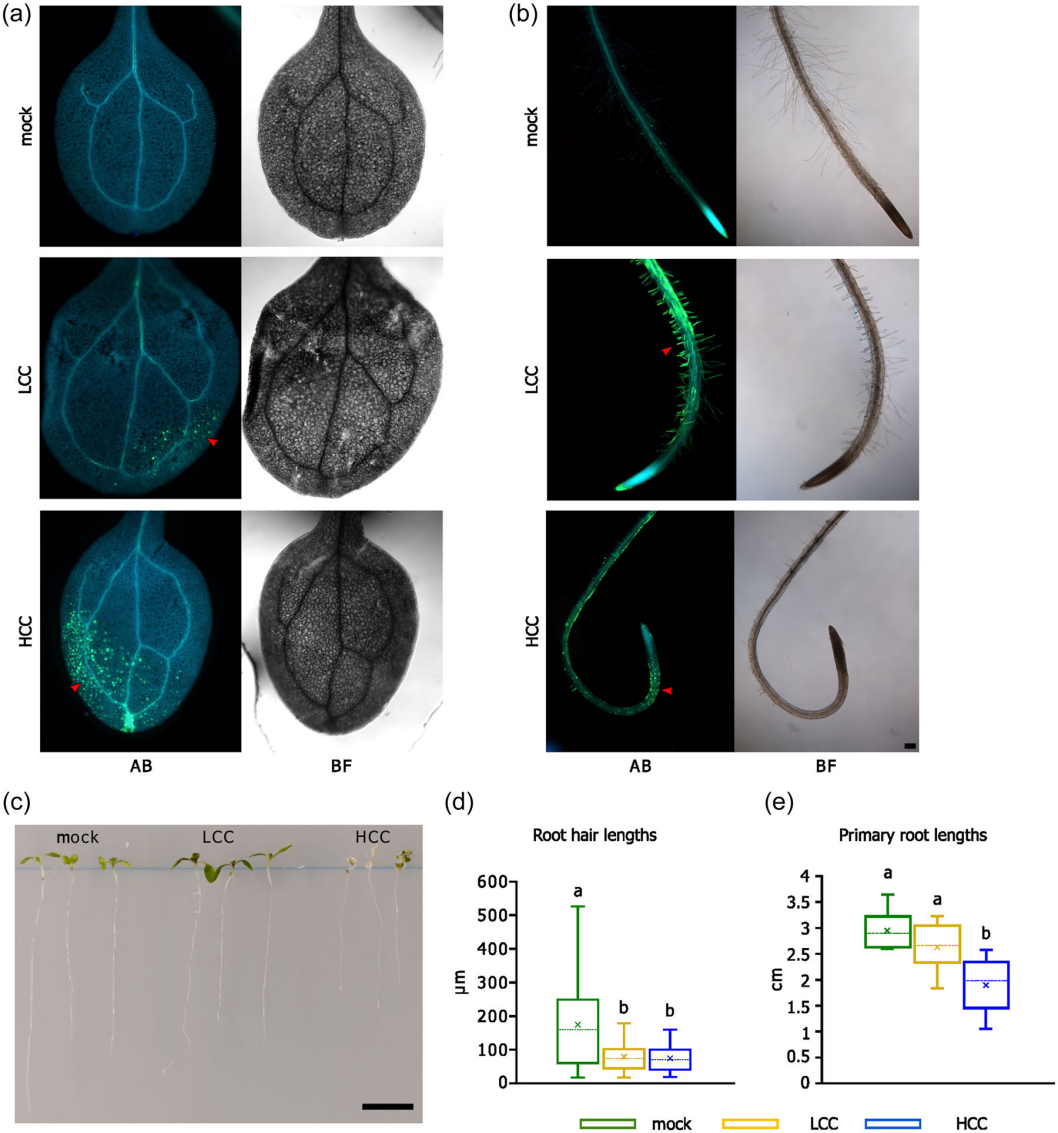
Concentration dependent modes of chitosan‐triggered callose deposition in seedlings. (a) Cotyledon leaves 24 h posttreatment (p. t.) with mock, low chitosan concentration (LCC), and high chitosan concentration (HCC) stained with aniline blue (AB) and according bright field (BF) appearance; red arrowheads point to yellowish callose signal. (b) AB and BF appearance of roots/root hairs 24 h p. t. (red arrowheads point to yellowish callose signals; bars = 100 μm). (c) Examples of appearance of seedlings with primary roots 24 h p. t. with mock, LCC and HCC (bar = 1 cm). (d) Root hair lengths 24 h p. t. with mock, LCC and HCC (*n* = 20–40). (e) Primary root lengths 24 h p. t. with mock, LCC and HCC (*n* = 8–11). Different small letters in (d) and (e) indicate significant differences between samples at *p* < 0.05.

When 5–7 day‐old *A. thaliana* seedlings transferred from solid to liquid medium were subjected to the two different concentrations of chitosan: a low 0.001% chitosan concentration (LCC), and a high 0.01% chitosan concentration (HCC), 24 h later, both treatments caused ectopic callose deposition in cotyledon leaves and roots (Figure 1a), appearing as a yellowish signal of anillin blue/sirofluor stained callose under the UV light. Specifically, LCC induced small callose deposits in leaves, without inhibiting primary root growth but with notable callose depositions in RH regions close to the root tips. On the other hand, high chitosan concentration (HCC) resulted in larger callose deposits in leaves, significant primary root growth inhibition, callose deposition in root epidermal cells but no callose encasement of RHs (Figure 1a,b).

Given the remarkable response of RHs to LCC, we investigated whether this effect was due to the particular chitosan composition. The commercially available chitosan is obtained from marine crustaceans' chitin by chemical deacetylation (up to 90%) with molecular weights (Mw) ranging from oligo‐ (<5 kDa) to polysaccharides (70–100 kDa). Therefore, the tests with the same low concentration with various chitosan variants of different molecular weight, viscosity, and solubility revealed a consistent effect on callose deposition along RH, confirming the chitosan specificity of RH callose deposition (Figure S1). Notably, non‐deacetylated chitin did not produce a similar response.

In conclusion, chitosan elicits specific responses in both above‐ground organs and roots and RHs, with outcomes dependent on the applied concentrations.

### 2.2 Callose deposition is PMR4‐dependent and uncoupled from the RH growth inhibition

**Figure 2:**
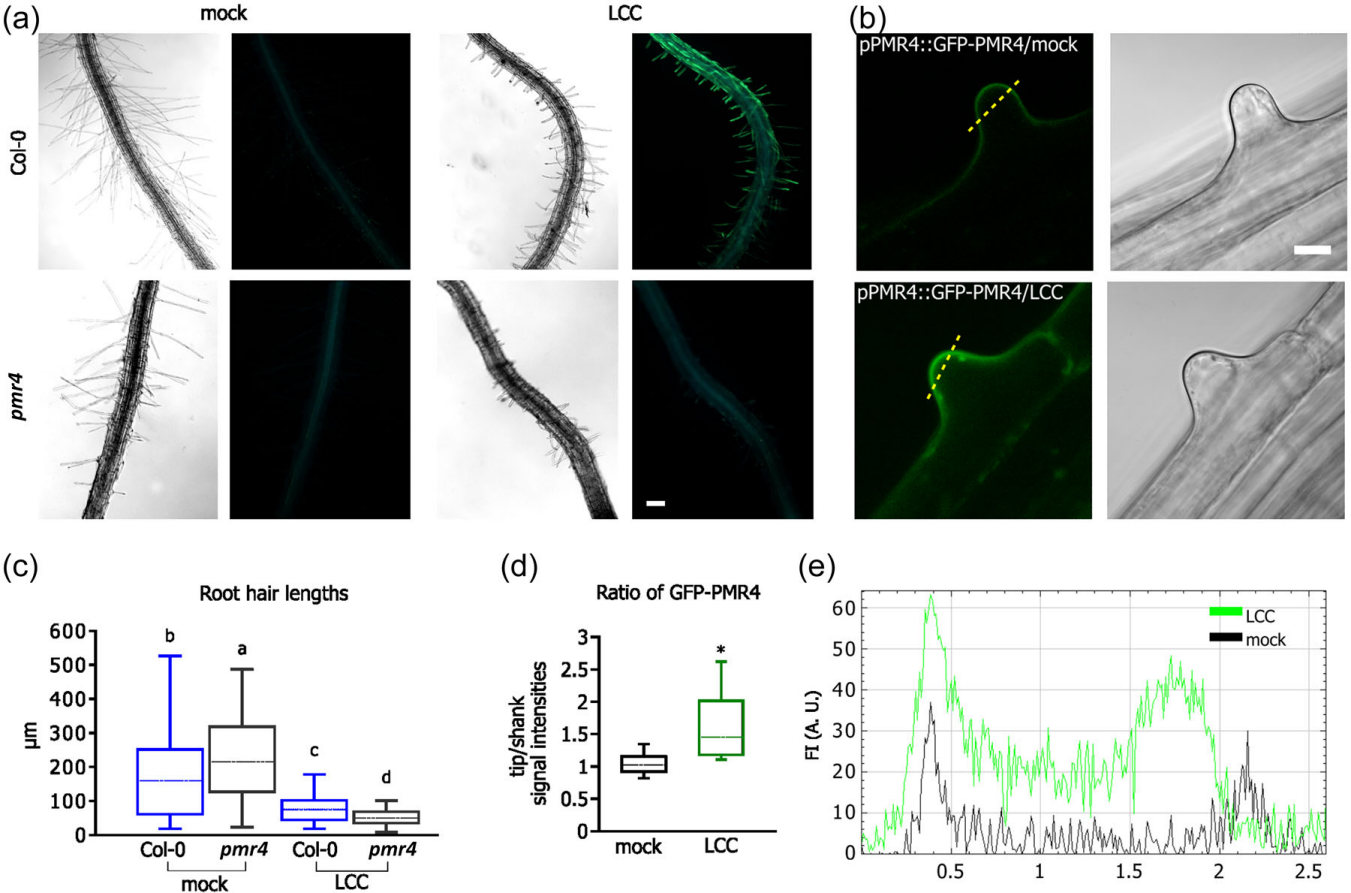
Callose deposition in LCC is PMR4‐dependent. (a) In contrast to the Col‐0 control, the *pmr4* mutant does not deposit callose along the root hair after the LCC treatment. (b) Upon the LCC treatment, at 4 h p. t. GFP‐PMR4 accumulates in the tips of growing RH, further confirming a role for PMR4 in RH callose deposition (left—GFP channel, right—bright field). (c) The RH growth is suppressed in the *pmr4* mutant to the similar extent as in the Col‐0 control, as shown by quantification of RH lengths. Small letters indicate significance of differences based on the ANOVA test (*n* = 100–200) for each line and treatment). (d) The ratio of GFP‐PMR4 tip/shank of RH signal intensities. The signal of GFP‐PMR4 is significantly more intense upon LCC treatment (*n* = 5–10 root hair cells for mock and LCC each). (e) The distribution of signal intensities along the sections marked in (b) for mock (black) and LCC (green) treatment. Bars = 100 μm in (a) and 10 μm in (b). LCC, low chitosan concentration. [Color figure can be viewed at wileyonlinelibrary.com]

To elucidate the key contributors to RH callose deposition, we examined RH modifications in the *gsl5/pmr4* mutant, lacking the callose synthase isoform responsible for pathogen‐induced callose deposition. The callose deposition induced by LCC was entirely abolished in the *pmr4* mutant (Figure 2a). Additionally, utilising the GFP‐tagged PMR4 construct (Huebbers et al., 2023), we observed a higher accumulation of PMR4 at the tip of newly growing RHs 4 h after chitosan addition compared to the mock treatment (Figure 2b,d,e). In addition to the *pmr4* mutant, we tested *cals8‐1*, a mutant in gene encoding the plasmodesmal callose synthase, as well as a range of other defence‐related mutants (including signalling coreceptor *bak1* and chitin receptor *cerk1*). However, all showed an RH callose pattern similar to that of WT/Col‐0 (Figure S2).

**Figure 3:**
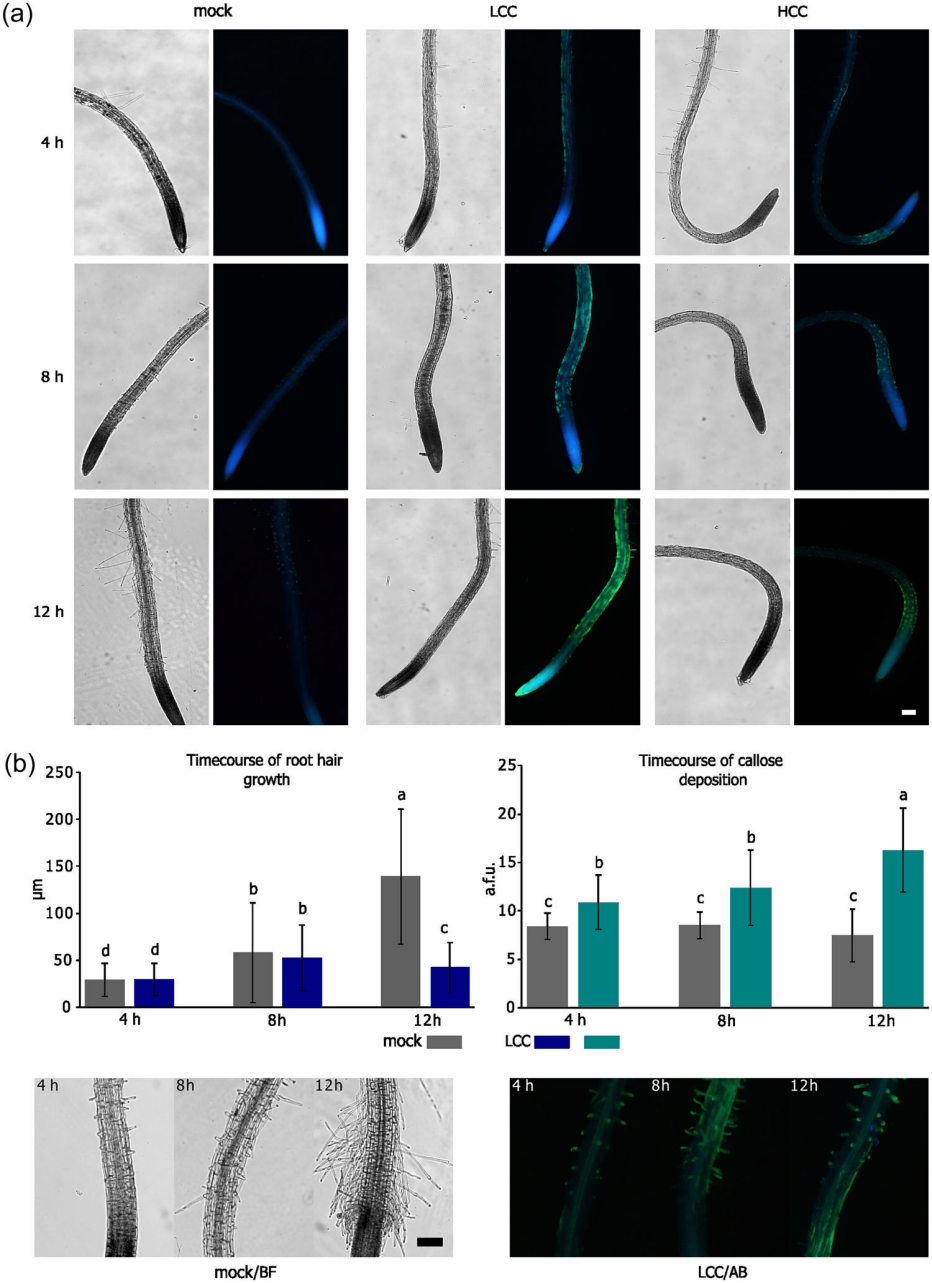
Time course of the decline of the RH growth and increase of the callose deposition in seedlings at 4, 8, and 12 h posttreatment (p. t.). (a) Examples of seedling appearance in indicated time points after the treatment with mock, LCC and HCC; always bright field on the left, and aniline blue stained roots on the right. (b) Quantification of root hair growth and callose deposition in indicated time points for mock and LCC. Graphs reveal significant changes between the mock‐ and LCC‐treated seedlings in the growth observable at 12 h p.t. (*n* = 90–140), and in the callose deposition already at 4 h p. t. (*n* = 20–35); both trends are illustrated by selected images on the left. Bars = 100 μm. AB, aniline blue; BF, bright field; HCC, high chitosan concentration; LCC, low chitosan concentration. [Color figure can be viewed at wileyonlinelibrary.com]

Given that LCC led to a decrease in RH growth, we hypothesised a potential connection with callose deposition and rigidification of the RH cell wall. However, the LCC‐induced RH growth decline in the *pmr4* mutant, which does not deposit callose, was similar to that in WT/Col‐0 (Figure 2c). We also monitored both callose deposition and root hair growth in WT after LCC addition, revealing that callose began to accumulate at the 4‐h time point, while the average inhibition of root hair growth became evident after 8 h, reaching significant differences from mock treatment approximately 12 h after chitosan addition (Figure 3). This temporal disparity in the pattern further confirms the independence of RH growth decline and callose deposition. In contrast to LCC, HCC treatment completely arrested the growth of new RHs already at the 4‐h time point (Figure 3).

In summary, LCC‐induced callose deposition in RHs is PMR4‐dependent and uncoupled from the mechanisms governing slower RH growth.

### 2.3 Chitosan LCC and HCC treatments trigger PTI‐related signalling with different intensities

**Figure 4:**
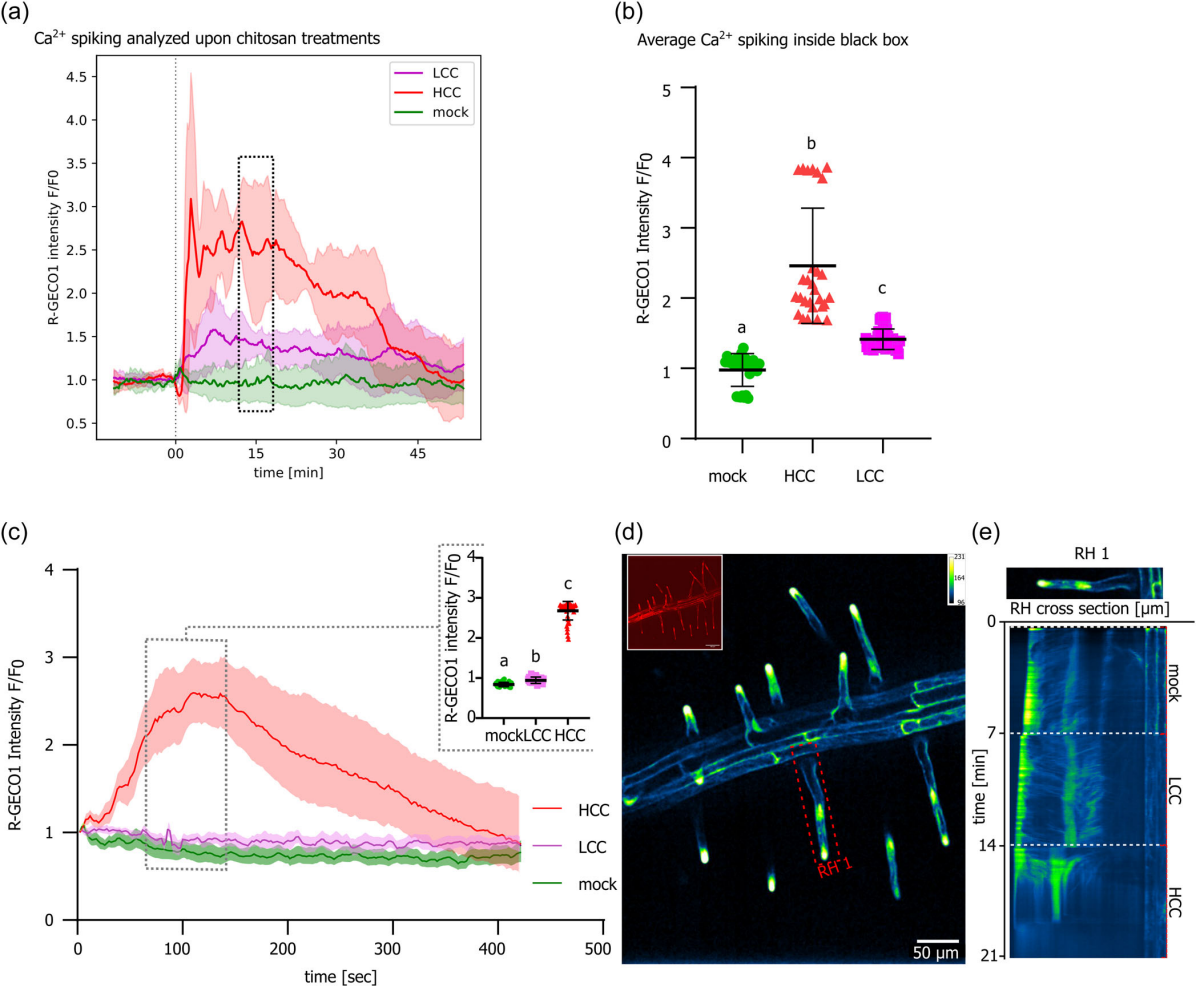
Chitosan‐triggered Ca^2+^ signalling in *Arabidopsis thaliana* seedlings. (a) Ca^2+^ spiking analysed as R‐GECO1 sensor fluorescence in root cells upon chitosan treatment shows slightly later onset with mild oscillations and long duration within the ca 45‐min frame of LCC treatment, while HCC induces prominent Ca^2+^ spiking with subsequent drop in signal intensity. The error envelope represents SD, *n* = 4–8. (b) Average Ca^2+^ spiking in selected time points (black box), LCC and HCC treatments, *n* = 20–50; small letters indicate significance of differences based on the ANOVA test. (c) Ca^2+^ spiking analysed as R‐GECO1 sensor fluorescence specifically in RHs upon mock or chitosan treatments (LCC, HCC) for 7 min. The error envelope represents SD, *n* = 20–30 RHs from at least four biological repetitions. Statistical analysis of the data highlighted by the grey inset box in panel (c). Plot shows single measured values with mean and SD error bar. ANOVA test with post hoc Tukey distribution was performed, *p* < 0.001. (d) Examples of R‐GECO1 signals in RHs as artificial LUTs (green fire blue); the calibration bar represents the signal intensity (box on the right). An example of the red signal appearance is shown in the upper left inset. (e) R‐GECO1 oscillations depicted via kymograph throughout the experiment (mock, LCC, HCC) of selected RH, indicated in panel (d) by the red dashed rectangle RH1. White dashed lines indicate the end of a particular subsequent treatment. HCC, high chitosan concentration; LCC, low chitosan concentration; RH, root hair. [Color figure can be viewed at wileyonlinelibrary.com]

We further wanted to uncover differences in general activation of early PTI responses between the LCC and HCC treatments. For that purpose, we first followed calcium spiking in roots using sensor R‐GECO1 construct (Keinath et al., 2015) in a 20‐min treatment of seedlings by the two concentrations of chitosan. There was only a mild Ca^2+^ increase in spiking in LCC treatment compared to HCC treatment, which triggered more prominent peaks of intracellular Ca^2+^ increase (Figure 4a,b, Figure S4A). Besides their intensity, the two treatments also differ in their patterns and peaking time, evidencing early specificity of the LCC‐ versus HCC‐induced responses. Interestingly, despite the obvious employment of the Ca^2+^‐permeable channels in chitosan sensing, *cngc2* and *cngc14* mutants have unaffected deposition of the callose to the RH cell walls, indicative of higher importance of the CNGC‐mediated signalling for the RH growth than for the callose deposition (Figure S3). This is in agreement with preserved capability of the *cngc14* mutant to react to LCC treatment with functional influxes; nevertheless, this peaking is significantly lower, evidencing cooperation of several Ca^2+^‐permeable channels during the chitosan sensing (Figure S4B)

Additionally, we assayed another parallel early signalling PTI pathway, MAPK activation, after the two chitosan concentrations 30‐min‐long treatments. Similarly, a western blot analysis of MAPK activation by phosphorylation (quantified as a ratio of band intensities obtained for the phosphorylated part and whole pool MAPKs) demonstrates significant MAPK activation in both cases. However, HCC causes a more prominent effect on the MAPK signalling pathway than the LCC (Figure S4C,D). The distribution of activation along the three tested MAPKs—MAPK3,6 and 4 was similar for both treatments. Accordingly, with these results, the pretreatment of seedlings with HCC, and not LCC, causes minor but significant enhancement of PTI, as demonstrated by the bacteria sensitivity and amplification assay upon seedlings inoculation with *Pseudomonas syringae pv. tomato* mutant in T3 secretion system *hrcC* (Pst *hrcC*; Figure S4E).

Having shown distinct fast calcium responses in roots after LCC and HCC treatment, we next analysed the spatiotemporal details of the Ca^2+^ reporter R‐GECO1 within the root hair (RH) cells. In mock treatment, the very tips of RHs undergo constitutive pulsatile changes of R‐GECO1 signal intensity; upon treatments, specific RH Ca^2+^ influx intensities and patterns are induced, especially in the case of HCC the prominent peaking is observable (Figure 4c–e; Video S1). This peak typically appears within 1–2 min at the RH tips, after which the signal propagates towards the interior of the RH cell and further spreads through the trichoblast towards other root cells (Video S1). In this experiment, the LCC treatment promoted a mild but statistically significant increase of R‐GECO1 fluorescence in comparison to the previous experiment (Figure 4a) where this difference was much higher. This mild reaction is likely attributable to the altered microscopic setup (see Section 5 for details).

These results confirm that both LCC and HCC treatment activates PTI‐related early responses, nevertheless, with different patterns and intensities. Importantly, our experiments also uncover the capability of RHs to activate Ca^2+^‐related signalling upon chitosan‐induced biotic stress.

### 2.4 Chitosan modulates endomembrane dynamics but not actin cytoskeleton in growing RHs

**Figure 5:**
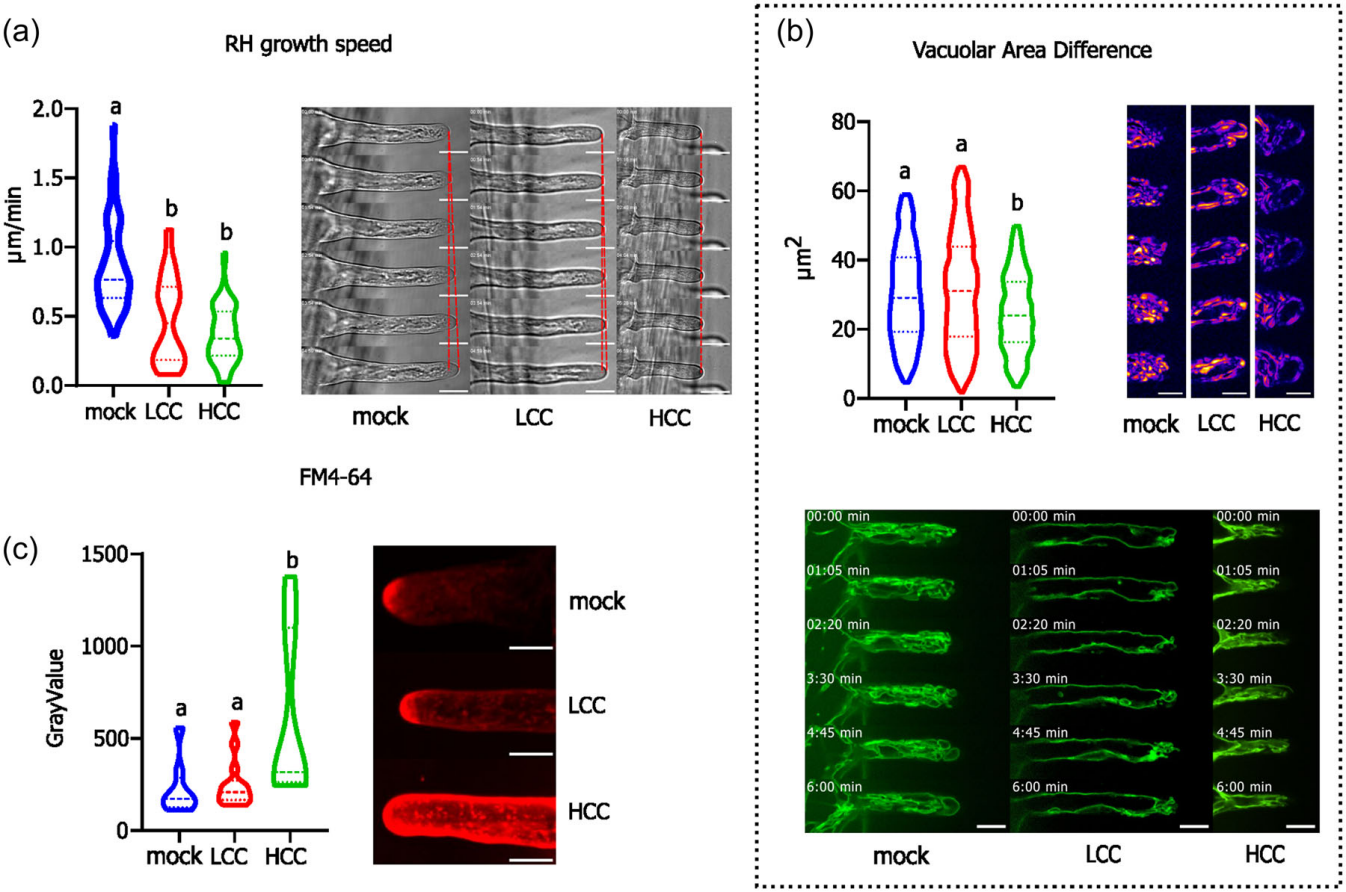
Subcellular dynamics during the early time points of LCC and HCC treatments. (a) The real‐time analysis of the RH growth during the mock, LCC, and HCC treatments based on the bright field images. The plot shows the RH growth rates for the mock, LCC and HCC treatments; small letters indicate significant differences based on Kruskal–Wallis test. (b) The quantification of the differential portion of the tonoplast visualised using PPase‐GFP between two consecutive frames as an area; examples of differential signals are shown in images on the right, the lighter coloration (pink) indicates higher differences than darker (violet) ones; examples of vacuolar dynamics are shown below. Small letters indicate significant differences based on the ANOVA test. Bar = 20 μm. (c) The analyses of the endocytosed FM4‐64 dye after 15 min of treatments for mock, LCC, and HCC. The endocytic compartment stained by FM4‐64 in the RH tip area is prominently enhanced in case of the HCC treatments. Small letters indicate significant differences based on Kruskal–Wallis test. On the right are examples of maximal intensity projections of FM4‐64 stained RHs. Bar = 10 μm. HCC, high chitosan concentration; LCC, low chitosan concentration; RH, root hair. [Color figure can be viewed at wileyonlinelibrary.com]

We then investigated if and how the chitosan‐triggered early signalling event affects the intracellular dynamics. We focused on well‐growing RH close to the root tip, and after applying mock, LCC and HCC treatments, we followed intracellular changes in localisation and dynamics of vacuolar and cytoskeletal genetic markers, as well as endocytic dye FM4‐64 in a real‐time approach (Figure 5). We monitored treated RH for 0–30 min. First, we observed an immediate and significant root hair growth cessation in the case of HCC‐treated seedlings, often accompanied by RH damaging and bursting. Additionally, with LCC treatment we also observed significantly lower average growth rates. Nevertheless, from the obtained data distribution, we could conclude that in the case of this treatment, RHs mostly either prominently slow down their growth, or they grow at rates similar to mock‐treated RHs (Figure 5a).

Interestingly, the vacuolar dynamics does not follow the trend found for growth and the RHs react to the two treatments contradictory—while LCC causes small and insignificant enhancement in vacuolar dynamics, HCC impairs the vacuolar dynamics in the RH tip area (Figure 5b). The cessation of vacuolar movement in the RH tip upon HCC treatment may be related to observation obtained for the same treatment on the enhanced FM4‐64 dye accumulation in RH endocytotic/vesicular compartment, likely as a consequence of collapsing endomembrane trafficking (Figure 5c; Figure S5A).

Since vacuolar membrane movements are facilitated by the actin cytoskeleton (Uemura et al., 2002), we also investigated the effect of the two chitosan concentrations on the organisation and dynamics of filamentous actin in RHs. Somewhat unexpectedly, there was no difference in the actin organisation during the early response to chitosan, as the dynamics of actin‐decorating fimbrin‐GFP were indistinguishable among the mock, LCC, and HCC treated plants (Figure S5B).

The microscopical observations thus confirmed that the LCC, despite causing prominent changes in CW composition and RH growth inhibition in later time points, does not impose a prominent stress that would disturb endomembrane compartments in the early time points. On the other hand, HCC treatment significantly inhibits RH growth, rapidly affects calcium gradient and oscillations in RH tip, blocks vacuolar dynamics and triggers accumulation of endocytotic/FM4‐64‐ marked compartment.

### 2.5 Chitosan induces changes in gene expression

**Figure 6:**
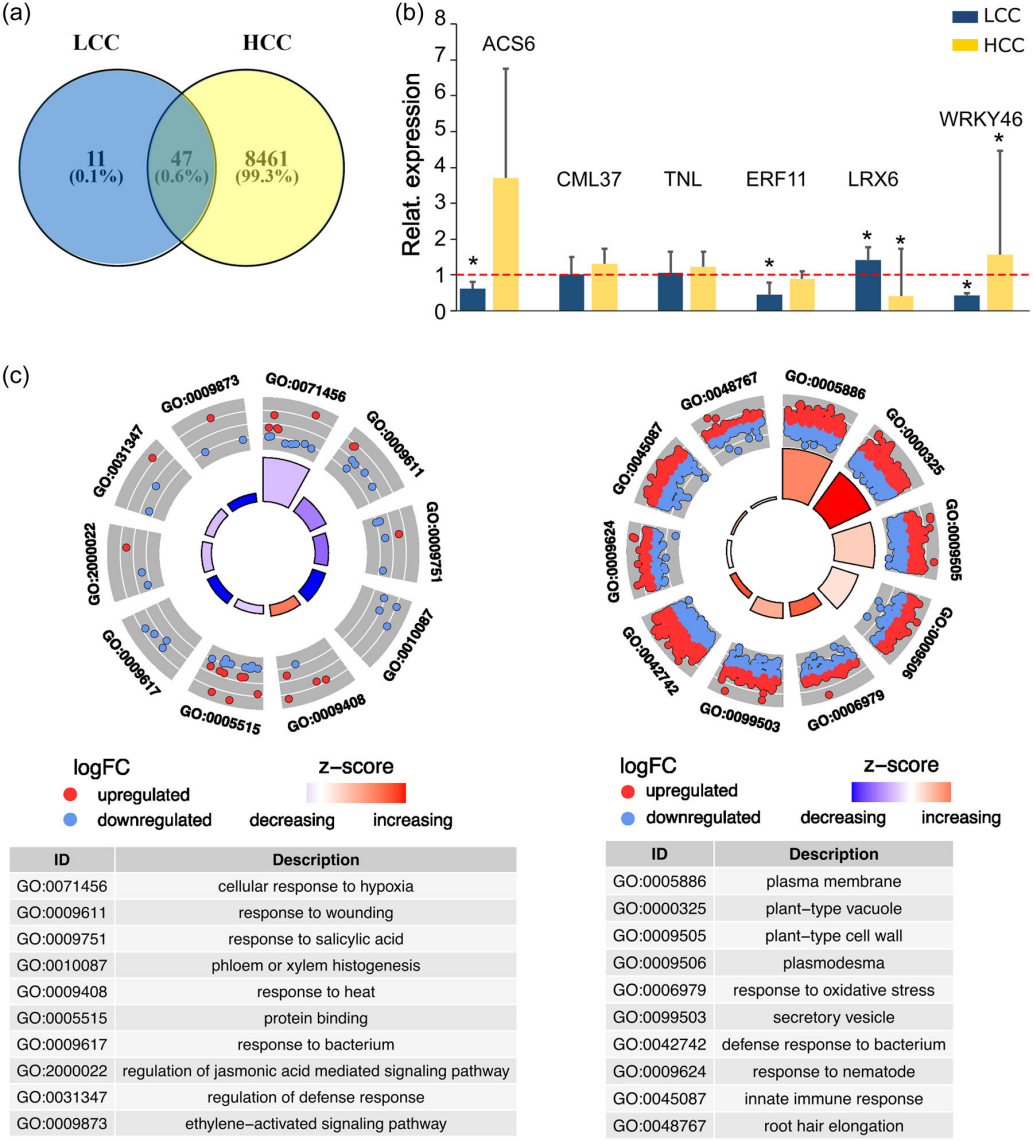
Chitosan induces changes in gene expression. (a) In RNAseq analysis, differentially expressed genes (DEGs), 58 for LCC, and 8507 for HCC were identified, (*q*‐val ≤ 0.05). (b) The differential regulation of several selected genes was confirmed by RT‐PCR, showing to be significant for four genes: ACS6, EFR11, LRX6, and WRKY46 (*n* = 3–7; mock expression levels were set as 1; significantly different levels of expression found for each of genes in LCC and HCC vs. mock, determined using *t*‐test, are marked by asterisks). (c) Circle plots showing 10 selected GO categories, which were found significantly enriched in LCC and HCC DEGs. The inner rings are bar plots showing the significance of the term deduced out of the *p*‐value (height) and the *z*‐score (gradual colour); the outer rings display scatter plots of the expression levels (logFC) for the genes in each term (related RNAseq and GO terms analyses are provided in Table S1). GO, gene ontology; HCC, high chitosan concentration; LCC, low chitosan concentration. [Color figure can be viewed at wileyonlinelibrary.com]

To obtain a broader perspective on chitosan responses and to gain further insights into distinct plant reactions to LCC and HCC treatment, we conducted RNAseq analysis of liquid medium‐grown Arabidopsis seedlings treated with mock, LCC or HCC for 24 h. The overall responsiveness of plants to LCC appears to be less pronounced compared to what is observed on the level of RHs, as only 58 differentially expressed genes (DEGs) were identified for LCC, and 8507 for HCC, for a *q*‐val ≤ 0.05 (*p*‐value corrected for false positives). Among the identified DEGs, particularly in the case of HCC, are participants of plant PTI responses (Figure 6a; Table S1). Eleven genes were specifically found to be differentially expressed under LCC treatment, with 10 genes downregulated and only one, AtLRX6 (Leucine‐rich repeat‐extensin 6; AT3G22800), upregulated. AtLRX6 belongs to the leucine‐rich repeat/extension‐type protein family with members previously reported to play a role in RH growth (Baumberger et al., 2003).

Additionally, we identified genes that were differentially expressed in LCC and HCC treatments; in contrast to HCC, which upregulates their expression, LCC downregulates these genes (the bottom five genes in Table 1). The differential regulation of several selected genes was confirmed by qRT‐PCR, showing significant differential expression for four genes: ACS6, EFR11, LRX6, and WRKY46 (Figure 6b).

**Table 1:** Changes in gene expression specific for LCC treatment.

gene identifier	gene name	protein function	fold change LCC	fold change HCC
AT1G72900	ERF11	ERF domain protein 11	0.49	insig.
AT2G22500	DIC1	uncoupling protein 5	0.59	insig.
AT2G46400	WRKY46	DNA‐binding protein 46	0.36	insig.
AT3G22800		leucine‐rich repeat (LRR)/extensin	1.85	insig.
AT3G56400	WRKY70	WRKY DNA‐binding protein 70	0.48	insig.
AT4G08950	EXO	phosphate‐responsive 1 family protein	0.70	insig.
AT4G11280	ACS6	1‐aminocyclopropane‐1‐carboxylic acid (ACC) synthase 6	0.47	insig.
AT4G24380		involved in 10‐formyltetrahydrofolate biosynthesis	0.42	insig.
AT5G41750		disease resistance protein (TIR‐NBS‐LRR class) family	0.42	insig.
AT5G59550		zinc finger (C3HC4‐type RING finger) family protein	0.63	insig.
AT5G64870		SPFH/Band 7/PHB domain‐containing, flotillin	0.49	insig.
AT1G72900		Toll‐Interleukin‐Resistance (TIR) domain‐containing protein	0.57	2.95
AT2G16660		major facilitator superfamily protein	0.52	1.30
AT2G34930		disease resistance family protein LRR	0.62	1.79
AT3G50930	BCS1	cytochrome BC1 synthesis	0.44	2.12
AT5G42380	CML37	calmodulin like 37	0.32	2.50

*Note*: Eleven genes (out of 58 DEGS; most of them with high RH expression) are specifically affected by LCC, when compared to HCC (out of 8508 DEGs); the last five genes are differentially expressed in LCC in comparison to HCC (fold change < 1 = downregulation, fold change > 1 = upregulation). Abbreviations: HCC, high chitosan concentration; LCC, low chitosan concentration.

When the identified DEGs were subjected to Gene Ontology (GO) term enrichment analysis, only several categories were found to be significantly enriched for the set of DEGs upon the LCC (Figure 6c; Table S1). In the case of HCC, both upregulated and downregulated genes showed enrichment in GO categories primarily related to endomembrane compartments, pathogen perception and immunity, cell wall modification, and root hair growth (Figure 6c).

The limited number of DEGs induced by LCC correlates with milder Ca^2+^ and MAPK response activation and with limited morphological changes observed mainly at the level of newly growing RHs. Nevertheless, DEGs found in HCC, congruently with these morphological changes, point to a prominent intersection of defence and endomembrane‐ and cell wall‐modification responses to chitosan.

### 2.6 Chitosan‐induced callose deposition corresponds with pectin methylation status

**Figure 7:**
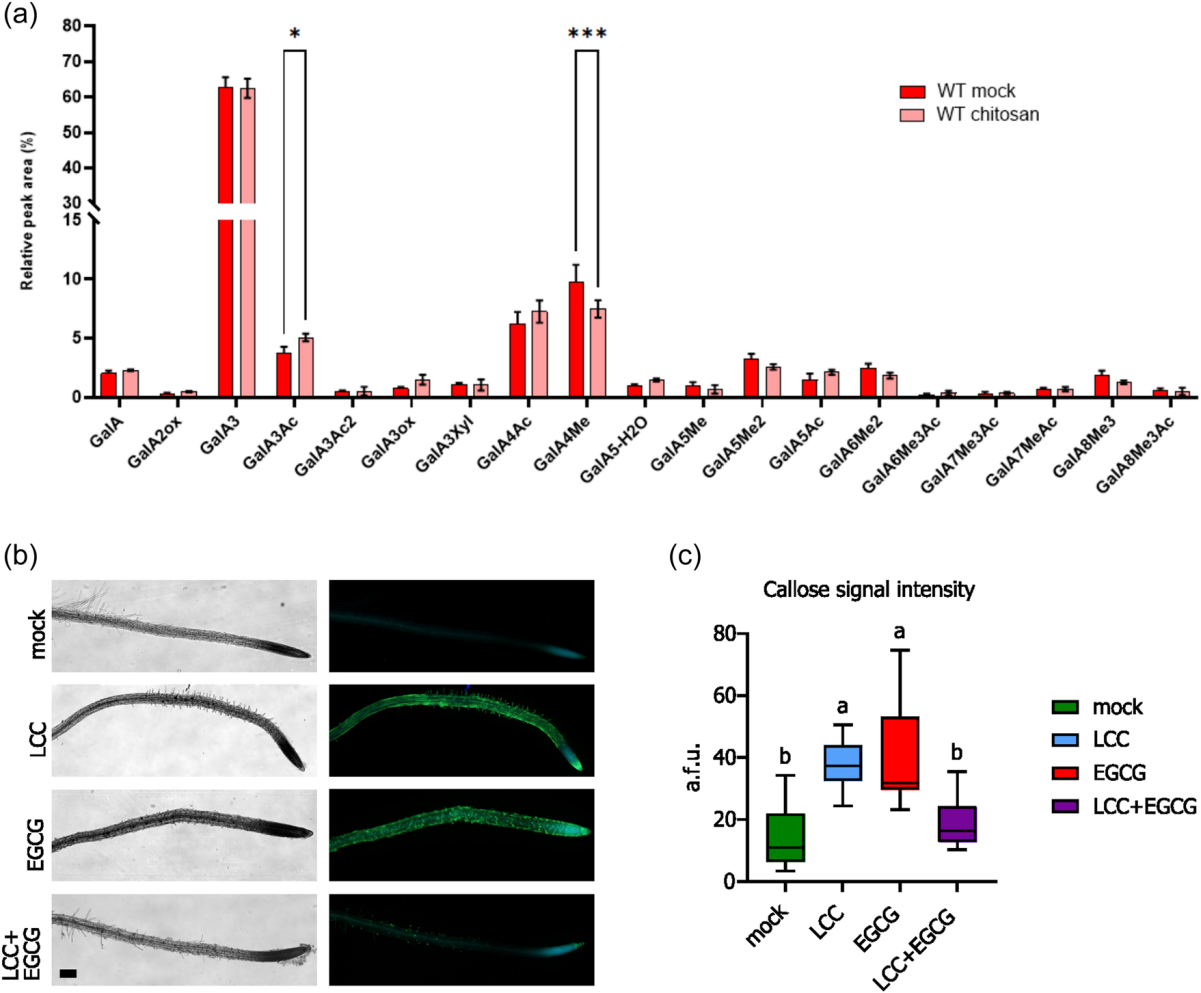
Chitosan affects pectin methylation status. (a) Enzymatic fingerprinting of homogalacturonans; the LCC treatment appears to trigger de‐methyl esterification and acetyl esterification, as evidenced by the significant decrease in relative amounts of GalA4Me and increase in GalA3Ac, respectively. Data represents mean ± SD, *n* = 4, *p < 0.05, **p < 0.01, student's *t‐*test. OGs are named GalAxMeyAcz. *x*, *y*, and *z* indicate the degree of polymerisation (DP) and the number of methyl‐ and acetylester groups, respectively. GalA: Galacturonic acid, Me: methylester group, Ac: Acetylester group. (b) When the seedlings are treated with the pectin methyl esterase (PME) inhibitor epigallocatechin gallate (EGCG), callose deposition in roots is induced, albeit in different patterns than after the LCC treatment. Nevertheless, simultaneous treatment with EGCG and chitosan appears to alleviate stress and slow down callose deposition (bar = 100 μm). (c) Quantification of callose signal upon LCC and/or EGCG treatments showing significant decrease in callose deposition upon simultaneous treatment (a. f. u. = arbitrary fluorescence units; *n* = 15 (three regions of interest [roi] of the same size from five roots/treatment); small letters indicate the significance of differences based on the ANOVA test). LCC, low chitosan concentration. [Color figure can be viewed at wileyonlinelibrary.com]

Considering the impact of chitosan on the expression of genes encoding for proteins with cell wall‐related functions, we examined if the chitosan, which is positively charged due to the protonation of glucosamine, can directly impact cell wall integrity and subsequently composition via ionic interaction with acidic cell wall components such as de‐methyl esterified homogalacturonan. For that purpose, the cell walls of the mock and LCC‐treated rosette leaves were analysed by the enzymatic fingerprinting of homogalacturonans using a commercial polygalacturonase (Voxeur et al., 2019). Oligosaccharides produced by enzymatic digestion were analysed by LC‐MS (Paterlini et al., 2022). The LCC treatment appears to trigger de‐methyl esterification and acetyl esterification, as evidenced by the significant decrease in relative amounts of GalA4Me and increase in GalA3Ac, respectively (Figure 7a).

To assess the significance of pectin methylation status in LCC‐induced responses in roots and RHs, we treated seedlings with the pectin methyl esterase (PME) inhibitor epigallocatechin gallate (EGCG) alone or in combination with LCC. We found that both treatments induce callose deposition, albeit in slightly different patterns. EGCG notably induces callose deposition along the more differentiated part of the roots (Figure 7b). However, simultaneous treatment with EGCG and chitosan appears to alleviate stress and slow down callose deposition, indicating that the effect of chitosan is at least partially attributed to deviations in pectin methylation levels (Figure 7c).

Given that inhibiting PME activity can disrupt RH callose deposition, we consider that the root hair callose deposition is a consequence of chitosan treatment‐induced demethylation of pectin, alongside activation of defence responses.

### 2.7 Callose deposition in RHs is conserved in other plant species

**Figure 8:**
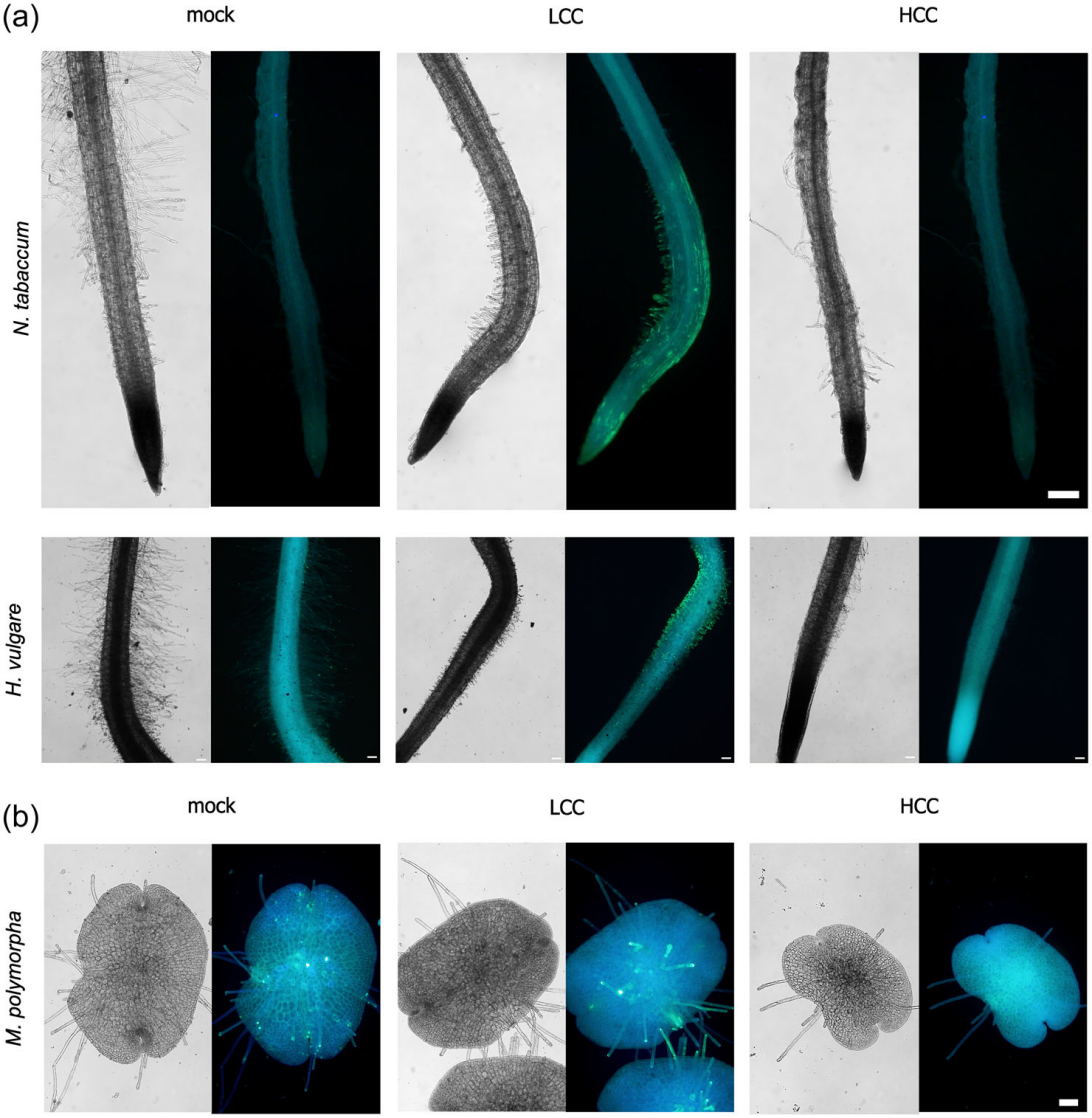
Callose deposition in RHs and RH‐like structures in other plant species. (a) Appearance of roots and callose deposition in RHs of *Nicotiana tabacum* (bars = 200 μm) and *Hordeum vulgare* (bars = 100 μm) upon mock and LCC and HCC treatments. (b) Responses to HCC and LCC including callose deposition are conserved in structures analogous to RHs, rhizoids growing out of gemma, in *Marchantia polymorpha*; bar = 100 μm. HCC, high chitosan concentration; LCC, low chitosan concentration. [Color figure can be viewed at wileyonlinelibrary.com]

We next tested whether the chitosan‐induced RH callose deposition is conserved beyond Arabidopsis. We first tested two model plants, a dicot from the asterid clade, *Nicotiana tabaccum* and a monocot, member of the grass family *Hordeum vulgare* (Figure 8a) and demonstrated that the callose encapsulation of RHs is preserved in these two species. In a similar experimental setup with more distal plant species, we were also able to induce the growth of the morphological structures functionally analogous to RHs, namely, rhizoids in the liverwort *Marchantia polymorpha*. When the plants were treated for 24 h with LCC, *M. polymorpha* rhizoids growing out of an asexual reproductive body gemma were enclosed by callose (Figure 8b). We further verified if the relation between the chitosan treatment and callose response could also be established for different plant cell model—regenerating protoplasts derived from the moss *P. patens* tip growing protonemal cells (Brejšková et al., 2021; Cove et al., 2009). We could indeed observe a prominent callose deposition caused by LCC, while HCC treatment had a deleterious effect on protoplast viability and regeneration (Figure S6A). Interestingly, in contrast to *P. patens* regenerating protoplasts, above‐ground tissue‐derived protoplasts of *A. thaliana* did not deposit callose while regenerating in the presence of chitosan (Figure S6B).

These results confirm that the potential of RH of various plant species and RH‐like structure to deposit callose is an evolutionary old intrinsic feature. However, this capacity may be exclusive for the tissue derived from the nonaerial plant parts, exhibiting fast cellular growth and newly generating the cell wall.

## 3 DISCUSSION

In this work, we uncovered that RHs have the ability to perceive chitosan as an elicitor, and depending on its concentration, to deposit callose into the cell wall and/or to inhibit RH growth. The two processes are not directly linked, and the two studied chitosan concentrations, the low (LCC) and the high ones (HCC), may induce plant responses that overlap in their quality and quantity, but also those which are specific. We also demonstrate that the shift in treatment stringency toward the milder concentrations of the stressor can unveil new defence‐ and development‐related phenotypes.

Unlike the majority of phytopathological studies, the LCC (0.001%), used in our study imposes a mild biotic stress, which young plants can tolerate (Aslam et al., 2009; Luna et al., 2011). The RHs are the most exposed cells which respond to LCC by slowing down their growth rate and by reinforcing the cell wall with callose. Callose deposition is typically described as an early defence response in leaf tissue, where it serves mainly to seal the plasmodesmatal connection between cells to prevent pathogen spread and nutrient withdrawal (Epel, 2009) or to prevent pathogens' entrance (Meyer et al., 2009; Ortmannová et al., 2022), but also in primary root epidermis after the defence‐eliciting treatments, displaying a similar spotty pattern as found in leaves (Millet et al., 2010). In agreement with this defence involvement, callose deposition is repressed in the establishment of mutualistic interactions (rev. German et al., 2023). Besides the defence‐related functions, callose also serves as a mechanical support in the new cell wall formation, in the pollen exine formation and germinating‐pollen plug, but also in a functional specialisation of trichome basal parts (Kubátová et al., 2019; Kulich et al., 2015; Ma et al., 2021; Motomura et al., 2022). The RH callose deposition observed in this work is tightly coupled with an immature state of the cell wall of the elongating root cells, and in the newly growing RHs in the zones closest to the root tip. Compiling our multifaceted results, including those on cell wall analysis showing altered state of pectin modification upon chitosan treatment, we hypothesise that the RH callose coating is the consequence of the two interconnected processes—of the elicitor‐caused defence signalling, as evidenced by the dependence on the typically pathogen‐induced isoform of callose synthase PMR4, but also of the newly formed cell wall modifications caused by chitosan interference with pectin methylation process by so far unknown mechanism. This is corroborated by the analysis of chitosan‐induced DEGs, which uncovered several pectin lyase‐like and methylesterase inhibitors, as well as PME2 (Table S2). The downregulation of PME2 is probably a compensatory mechanism due to increased portions of demethylated pectin regions. Notably, these RNAseq results are in agreement with results obtained from seedlings co‐treated with chitosan and EGCG, and with general importance of pectins and callose in cell wall build‐up and regeneration (Dehors et al., 2019; Okada et al., 2024). Further assessment is needed to understand how the physical features of chitosan and pectins—such as their ability to form interpolymer hydrogels (Morello et al., 2023)—as well as other cell wall components, influence the RH responsiveness to chitosan.

Interestingly, the LCC treatment did not affect the growth of young plants or their further development, suggesting an adaptation to these LCCs, which is also evident in the dampening of early phases of Ca^2+^ spiking patterns. In our real‐time imaging experimental conditions (Figure 4), less intense R‐GECO1 reaction is observed, compared to the one observed in the setup using microfluidic chip, probably as a consequence of the agar block‐absorbed or buffered portion of the LCC treatment solution. Nevertheless, LCC stimuli are perceived by other root parts and above‐ground organs, which is confirmed also by MAPK assays.

Unlike LCC, the HCC treatment has a prominent effect on the RH and root cells viability, morphology and growth, and on the subcellular level also on appearance and subsequent loss of complexity of the RH tip‐localised portion of the vacuolar compartment. The HCC causes the vacuolar withdrawal and dismission of the complex tubular tip‐localised vacuolar growing parts. Given the massive uptake of FM4‐64 dye in HCC‐treated RHs, this may be the consequence of the enlargement of the endocytic zone in the RH tip. Despite the critical importance of the actin cytoskeleton in both RH growth and responsiveness to pathogens, in our experiment, at an early time points, there was no significant modification of fimbrin‐GFP‐labelled actin filaments upon chitosan treatments (Figure S5, see also [Porter & Day, 2016; Ringli et al., 2002; Sun et al., 2021; Vaškebová et al., 2018]). It remains to be elucidated if the microtubules could play a more important role in observed modification of cell wall composition and growth, similarly to what has been found for the tobacco pollen tube (Cai et al., 2011).

The RNAseq data from LCC‐ and HCC‐treated seedlings corroborate the existence of primarily quantitative differences between the two treatments since only a minority of DEGs was found to be specific for LCC. Despite the scarcity of the LCC‐specific candidates, we consider that the role of one of the identified DEGs, LRX6 is worthy of further inspection, since related pollen tube‐expressed isoforms are involved in the pollen tube cell wall pectins and callose deposition (Wang et al., 2018). Notably, LRX6 ortholog LRX1 was recently shown to regulate the deposition of pectins in Arabidopsis RHs (Schoenaers et al., 2024).

The LCC/HCC treated RH of tobacco and barley plants, as well as rhizoids of *M. polymorpha*, which despite their evolutionary unrelated origins provide the same morphological and subcellular responses as RHs, imply strong evolutionary conservation of this response that probably resides in the employments of the evolutionarily conserved callose synthases (Ušák et al., 2023). These mechanisms conserved in independently‐evolved RHs and RH‐like structures terrestrial plants might have an origin in the callose contribution to the newly formed post‐meiotic cell wall of spores, but they may have also been involved in the onset of responses of plant cells to the hostile environmental conditions of land/soil, also including pathogen threat (Herburger & Holzinger, 2015; Ks et al., 2020).

In addition to demonstrating the potential of mild chemical/eliciting treatments of plants for opening new perspectives in plant studies, our work also highlights the usefulness of a simple experimental setup based on a single, exposed, and sensitive cell. This approach could be further employed for gaining the knowledge of the root and RH‐related immunity, as well as in the studies of the overall plant fitness in relation to the rhizosphere conditions.

## 4 CONCLUSIONS

The effect of the low concentration chitosan treatment on Arabidopsis RHs, and similar effect in other plant species, expose their unexpected deeply rooted capacity to react by the callose accumulation in response to this fungal elicitor. The root hair cell wall callose deposition is PMR4/GSL5‐dependent and occurs in parallel but independently of the root hair growth inhibition. The higher chitosan concentration treatments cause prominent root hair growth inhibition that precludes the callose deposition, leading to the phenotypic difference between the two treatments; this intensive root hair growth inhibition is probably the consequence of the emphatic defence alerting, as well as interference with the root tip‐localised vacuolar and endocytic compartments.

## 5 MATERIAL AND METHODS

### 5.1 Plant cultivation

For seedlings and plants cultivation, seeds were surface sterilised (5 min in 70% ethanol, 2 × 5 min in 10% commercial bleach, rinsed three times in sterile distilled water) and stratified for 2–3 days at 4°C. Seeds were then germinated and grown on vertical ½ MS agar plates (half‐strength Murashige and Skoog salts, Duchefa Biochemie, supplemented with 1% sucrose, vitamin mixture, and 1.6% plant agar, Duchefa Biochemie) at 21°C and 16 h of light per day for 5–7 days. For propagation of plants, seedlings were transferred into Jiffy Products International pellets and grown at 22°C and 16 h of light per day in growth rooms.

The following previously published Arabidopsis mutant and transgenic lines were used in this study: *bak1‐4* (CS71777; [Kemmerling et al., 2007]), *cals8‐1* (SALK_037603; [Cui & Lee, 2016]), *cerk1‐1* (pst14772; [Miya et al., 2007]), *cngc14‐1* (SALK206460; [Shih et al., 2015]), *cngc14‐2* (WisDsLox437E09; [Shih et al., 2015]), *cngc2‐3* (SALK_066908; Chin et al., 2013), *pmr4‐1* (CS67159; [Vogel & Somerville, 2000]), *rbohD‐3* (N9555, Torres et al., 2002 11756663), *cngc14‐2*xGCaMP3 (Qi et al., 2023; Toyota et al., 2018), promPMR4::GFP‐PMR4 (Huebers et al, 2023), fimbrin‐GFP (Wang et al., 2004), and vacuolar pyrophosphatase (PPase) VHP‐GFP (gifted by Karen Schumacher, Heidelberg). The wild type (WT) Col‐0 was used as control. When needed, primers used for the genotyping were the same as used in the above‐cited original studies.

### 5.2 Chitosan treatment

Plants were propagated in vitro for 5–7 d on vertical plates with ½ MS solid medium, and then transferred using forceps into liquid ½ MS (control) or liquid ½ MS with chitosan added as 100x and 1000x diluted stock solution. Stock solution was prepared as 1% chitosan (Merck, Cat. No C3646) in 1% acetic acid, and subsequently filter sterilised. Usually 4–8 plants were treated in 3 mL/well, in 6‐multiwell plates (Nunclon, Thermo Fisher Scientific). Plants were cultivated for 24 h 16/8 h light/dark at 21–22°C without shaking. Additional verifications confirmed that there were no differences between the mock treatment with and without corresponding dilution of acetic acid. Several other chitosans used for analyses were purchased from Merck, Germany (Cat. Nos 523682, 448877, and 419419), as well as chitin (C7170). The chitin stock solution was prepared as described in Millet et al. (2010). Briefly, 10 mg/mL of chitin was dissolved in water, the solution autoclaved and centrifuged, and supernatant saved as a stock solution.

Comparison of chitosan and PME inhibitor (−)‐epigallocatechin gallate (EGCG; E4268, Sigma‐Aldrich) treatments was performed in liquid ½ MS, 4–8 plants were treated in 3 mL/well, in 6‐multiwell plates. EGCG treatment was performed according to Mravec et al., 2014. The stock solution 20 mM was prepared by dissolving EGCG in ½ MS, and added to seedlings 1000× diluted alone or in combination with chitosan. Plants were cultivated for 24‐h 16/8‐h light/dark at 21–22°C without shaking and subsequently subjected to further analyses.

### 5.3 Callose staining

After 24 h of incubation, plants were transferred to 3 mL of ethanol for 6–24 h at room temperature for destaining, with mild shaking, and subsequently transferred into 3 mL of 150 mM K_2_HPO_4_. After 1 h, aniline blue (Cat No 8142 Lachema and 415049 Sigma‐Aldrich; final concentration 0.005% solution in 150 mM K_2_HPO_4_) was added and seedlings stained for additional 24 h with mild shaking and observed under the Olympus BX51 microscope with attached DP74 camera and B‐X‐UCB controller (Olympus; Evans et al., 1984). For visualisation of aniline blue/sirofluor stained callose we used fluorescent lamp ULH100HGAPO with fluorescent burner URFLT200, with DAPI filter settings, and objective 5x or 10x. Quantification of callose signal intensity was performed using 2–3 regions of interest (ROIs) of the same size for 5–10 roots for each treatment/line using ImageJ Fiji (Schindelin et al., 2012).

### 5.4 Root and root hair phenotype examination

The plant responses to pathogens were monitored by microscopy and documented by photography. The seedlings were placed on microscopic glass, their primary root stretched and lengths measured. Microscopic analysis of root tips and RHs was performed using an Olympus BX51 microscope with attached DP74 camera and BX‐UCB controller (Olympus). The camera was operated by CellSoft software (Germany). For each treatment/genotype 5–10 plants were photographed and analysed. Root hair lengths were determined for the first 20–40 RHs starting from the root tip for each plant and used for statistical analysis.

### 5.5 Ca^2+^ signalling reporter imaging and evaluation

*A. thaliana* seedlings expressing R‐GECO1 Ca^2+^ signalling reporter were vertically grown in a growth chamber at 23°C by day (16 h), 18°C by night (8 h), 60% humidity and light intensity of 100 μmol photons m^−2^s^−1^ (Keinath et al., 2015). Microfluidics experiments were performed using 4‐day‐old seedlings placed into a microfluidic chip and enclosed by a cover glass (Serre et al., 2021). Channel one contained a control solution (½ MS, fluorescein dextran 6 μg/mL), while channel two contained a treatment solution (½ MS, 0.01%, or 0.001% chitosan, HCC and LCC, respectively). The media flow rate was 3 μL/min (OBI1, MFS2 Elveflow, and Elveflow software ESI (v.3.04.1)). The system was mounted to a vertical microscope stage and kept for 15‐min plant recovery. The media exchange was indicated by fluorescein‐dextran signal intensity. For *cngc14‐2* background another reporter GCaMP3 (Qi et al., 2023; Toyota et al., 2018) was used.

Plants were imaged using a vertical stage Zeiss Axio Observer 7 with a Yokogawa CSU‐W1‐T2 spinning disk unit with 50‐μm pinholes equipped with a VS‐HOM1000 excitation light homogeniser (Visitron Systems), objective Zeiss Plan‐Apochromat ×10/0.45. Images were acquired using VisiView software (Visitron Systems, v.4.4.0.14). R‐GECO1 was excited by a 561 nm laser, and fluorescein dextran by a 488 nm laser. The GCaMP3 was excited by a 488 nm laser. The signal was detected using a PRIME‐95B Back‐Illuminated sCMOS camera (1200 × 1200 pixels; Photometrics). The seedlings were imaged every 13 s for 60 min, for GCaMP3 and *cngc14‐2*xGCaMP3, every 20 s for 30 min. The signal intensity of R‐GECO1 was measured inside the root at the root late elongation zone using ImageJ Fiji software (Schindelin et al., 2012). The intensity of the background was measured in areas not affected by the root response and subtracted from the R‐GECO1 values. The intensity of fluorescein‐dextran was measured outside of the root. The data were normalised using division by initial fluorescence intensity values (*F*/*F*0). As the initial value *F*0, we used the average fluorescence intensity at five‐time points before treatment.

### 5.6 Confocal and real‐time microscopy of RHs

Confocal and real‐time imaging was done on the Spinning disc (SD) microscope Nikon (Eclipse Ti‐E, inverted) with Yokogawa CSU‐W1 SD unit (50 mm) equipped with Omicron LightHUB ULTRA light source, Plan‐Apochromat L 20×/0.75 or Plan‐Apochromat LS 40×/1.25 WI objectives were used for capturing. Microscope is operated by NIS Elements 5.30 software. GFP was observed using a 488 nm excitation laser with Semrock brightline Em 525/30 filter. FM4‐64 was observed using a 561 nm excitation laser with Semrock brightline Em 641/75 filter.

Small agar block with in vitro cultivated 6‐day‐old seedlings was gently cut from the cultivation plate and transferred into sterile microscopic chambers containing 100 μL of liquid ½ MS. Chambers were covered with lids and left overnight in a cultivation chamber. For real time treatments we used custom‐designed 3D printed perfusion add‐on which fits into the microscopic chamber and allows exchange of media inside the microscopic chamber during microscopy session. To mediate the exchange we used medical grade silicone tubes mounted onto a peristaltic pump and perfusion add‐on with speed approximately 100 μL/min. All microscopic samples were 10 min pretreated with the mock solution right before capturing, chitosan treatment was applied directly during the microscopic session. For mock treatments, the liquid ½ MS was used. For chitosan treatments, 0.001% chitosan (LCC) or 0.01% chitosan (HCC) were added into the ½ MS. For the FM4‐64 (Thermofisher scientific) staining we used the same perfusion settings and solutions just with the addition of 1000× diluted FM4‐64 dye (1 mM stock concentration). Samples were treated for 15 min before observation.

Since the vacuole of RHs is very dynamic and complex, we did not analyse vacuolar size itself, instead, we analysed how much the vacuole changes its shape between consecutive frames. To achieve this the *stack difference* command inside *multi kymograph* plugin ImageJ Fiji followed by analysis of *moments* thresholded area of the result image was used. See supporting information *VD_macro.ijm*.

For the FM4‐64 uptake analysis the average Z projections of single RH were taken with subsequent analysis of signal intensity within inner cell space (ImageJ Fiji). Actin dynamics was analysed using the same procedure but with a longer gap between consecutive frames (10 s).

For RH R‐GECO1 signal intensity analyses the same microscopic perfusion add‐on was used, using a 561 nm excitation laser with Semrock brightline Em 641/75 filter. The 5‐6 day old RGECO1 expressing seedlings were transferred into microscopic chambers, covered with a lid and kept overnight in the cultivation room. Samples were treated using the same chitosan solutions as in previous experiments (LCC, HCC). Roots were captured every 1 s for 7 min for each part (mock, LCC, HCC) in the case of LCC and HCC, we added 30 s long (capturing time one frame per 0.1 s) to monitor fast changes. These superfast scanning parts were removed for the figures.

The PMR4‐GFP was observed using Zeiss LSM880 confocal microscope with 63× oil immersion objective, and recommended excitation and filters setting for GFP. The images were analysed using Zen 2.1 Software (Carl Zeiss GmbH) and ImageJ Fiji. The signal intensity was quantified using regions of interest (ROIs) positioned on the plasma membrane root hair tip area and shank area, for 5–10 RHs, 4 h after the mock or LCC treatment.

### 5.7 Protein extraction, Western blot and MAPK assay

Total protein extracts were isolated from 5 to 7‐day‐old *A. thaliana* seedlings according to procedure described in (Fernandez & Beeckman, 2020). Briefly, untreated and chitosan treated tissue samples were ground in liquid nitrogen and dissolved in an extraction buffer containing Phosstop (Roche), and the concentration of proteins determined by the Bio‐Rad protein assay kit with bovine serum albumin (BSA) as the standard. The extracts were denatured by boiling in a 6× SDS loading buffer. The protein samples were separated by 10% SDS‐PAGE and analysed by Western blot using the *α*‐p44/42‐ERK antibody (SAB4301578), anti‐MAPK3 (M8318, Sigma), anti‐MAPK4 (A6979, Sigma), and anti‐MAPK6 (A7104, Sigma). The primary antibodies were incubated with the membranes for 3 h at room temperature in the blocking solution. Horseradish peroxidase‐conjugated antibodies (anti‐rabbit and anti‐mouse; Promega) were applied followed by chemiluminescent ECL detection (Amersham) by the Bio‐Rad documentation system. Using the Gel Analysis function of ImageJ, signal intensities for protein bands were determined for each treatment from three different samples. Loading consistency was examined by staining the membrane with Ponceau S.

### 5.8 Bacterial assay

Bacteria susceptibility assay was performed according to Ishiga et al. (2011) and Pečenková et al. (2020) with modifications. Briefly, plants grown in vitro (½ MS, at 21°C, 12/12 h of light/dark per day, for 6 days) were transferred to liquid ½ MS in 6‐well plates, and pretreated with mock, or with chitosan (LCC and HCC). After 24 h, the medium was exchanged, and the *P. syringae pv. tomato hrcC* (Pst *hrcC*) OD = 0.01, was used for seedlings inoculation. After the 24 h incubation with mild shaking 50 rpm, ten seedlings in one sample, always three samples for each treatment, in two replications) were homogenised, homogenates subsequently diluted in series, plated out, and after approximately 24–36 h of incubation on 28°C colonies counted.

### 5.9 RNA isolation and RNAseq analysis

RNA was isolated from young, 6–7‐day‐old whole seedlings (transferred from vertical ½ MS plates to liquid ½ MS for treatments), from three independent samples (each ca 80–100 mg [ca 40–50 seedlings]), using the RNeasy Plant kit (Qiagen) according to the manufacturer's instructions. Isolated RNAs were stabilised by GenTegra technology microtubes (GenTegra, Pleasanton, California, USA). Strand‐specific cDNA libraries were constructed from polyA‐enriched RNA and sequenced on the Illumina NovaSeq 6000 platform with subsequent analysis performed by Eurofins. The RNA‐seq data used in this study are deposited in the National Center for Biotechnology Information Gene Expression Omnibus database under the accession number GSE239633.

Gene Ontology (GO) Enrichment analysis for DEGs (*q*‐value determined by sleuth R tool lower or equal to 0.05) was done in DAVID (https://david.ncifcrf.gov/, Huang et al., 2007). Visualisation of results of GO analysis was done using the GOplot tool in R (Walter et al., 2015).

### 5.10 Reverse transcription and quantitative PCR

The Q‐RT‐PCR was performed according to Müller et al., 2021. Briefly, approximately 1 µg of DNAse‐treated RNA was reverse‐transcribed using M‐MLV reverse transcriptase, RNase H‐, point mutant (Promega). Quantitative PCR was performed using GoTaq qPCR Master Mix (Promega) at 58°C annealing temperature on a LightCycler480 instrument (Roche). PCR efficiency was estimated using serial dilution of template cDNA. Positive transcript levels and the quality of PCR products were verified by melting curve analysis. The primer sequences for control genes and selected LCC‐specific genes are shown in Table S3.

### 5.11 Cell wall preparation and enzymatic fingerprinting

For cell wall analysis plants have been propagated in Jiffy pellets for 5–6 weeks, 10/14 h of light/dark regime. Plants were treated by dipping into the mock (distilled water with 0.005% Silwet) or LCC chitosan (distilled water with 0.005% Silwet and 0.001% chitosan), and left for 24 h in the growth chamber. Discs from treated and nontreated leaves were submerged in 96% (v/v) ethanol and boiled at 70°C for 10 min. The pellets were collected by centrifugation (13 000 g for 10 min) and dried in a speed vacuum concentrator at 30°C overnight. Samples were digested with 1 U/mg DW sample of *Aspergillus aculeatus* endo‐polygalacturonase M2 (Megazyme, Bray, Ireland) (Paterlini et al., 2022) in 50 mM ammonium acetate buffer (pH 5) at 37°C for 18 h. Samples were centrifuged at 13 000 rpm for 10 min and 100 µL of the supernatants were transferred into vials. The oligosaccharides released from digestion were analysed according to (Paterlini et al., 2022).

### 5.12 Comparative root hair callose deposition assay

*Nicotiana tabacum* and *Hordeum vulgare* plants were cultivated, treated and analysed for RH callose deposition the same way as *A. thaliana* plants. The liverwort *Marchantia polymorpha* Tak‐1 has been maintained on ½ Gamborg media ([Gamborg et al., 1968]; G0210 Duchefa) supplemented with 1.2% agar under standard conditions (22°C, 16/8 light/dark, ca. 180 μmol/m^2^/s). Chitosan treatments were done with gemmae analogously to an *A. thaliana* procedure, with a slight change, where the liquid ½ MS has been replaced with ½ Gamborg media supplemented with 1% sucrose to enhance the rhizoids initiation.

### 5.13 Protoplasts preparation from physcomitrium patens and *Arabidopsis thaliana*

The moss *P. patens* Gransden strain was routinely propagated in vitro on BCD medium supplemented with ammonium tartrate dibasic (BCDAT), according to (Cove et al., 2009), in a climate chamber (16 h of light/8 h of dark, 25°C, illuminated by fluorescent tubes at 50–70 µmol m^−^^2^s^−^^1^). Protonemal tissue (6–7 days after propagation) from cellophane‐grown culture was transferred into the 1% Driselase/8% mannitol solution. When filamentous structure became invisible (approx. 1 h at RT), protoplast suspension was filtered via/through 100 µm steel mesh. Protoplasts were diluted in 8% mannitol up to 10 mL and spun down (700 rpm for 4 min, without brake) and this step was repeated three times. Protoplast sediment was carefully resuspended between centrifugations to avoid aggregation of protoplasts. Finally protoplasts were incubated in the liquid regeneration medium (BCDAT + 5 mM NH_4_ + 6% w/v mannitol + 10 mM CaCl_2_) overnight at RT.

Arabidopsis protoplasts were prepared according to Yoo et al. (2007). Briefly, Arabidopsis leaves from 3 to 4 weeks old plants grown under short day conditions (8/16 h light/dark) were cut onto 2 mm stripes. The 2 g of prepared plant tissue were submerged in 37°C warm protoplast solution (0.4‐M mannitol, 1.5% cellulase, 0.4% macerozyme, 20 mM MES pH 5.7, 20 mM KCl, 0.1% BSA, 10 mM CaCl_2_) and vacuum has been applied for 30 min. The submerged leaves were kept in the dark in RT. After 3 h, the flasks with protoplasts were gently swirled and the quality of protoplasts was checked with a microscope. The protoplast solution was washed with a WI buffer (4 mM MES pH 5.7, 0.5 M mannitol, 20 mM KCl), filtered through miracloth and clarified with multiple centrifugations (3 × 100 g, 2 min). For the recovery of protoplasts, 0.7 mL of WI solution in a 12‐well plate was applied. Both types of protoplasts were left to recover for 3 h, and were subsequently treated with chitosan or mock for 24 h. Protoplasts were stained with aniline blue and the final condition of protoplasts monitored with the microscope Olympus BX51 microscope with attached DP74 camera.

### 5.14 Image analysis and statistics

Image processing software was employed for image data quantification. Root and root hair sizes were analysed using AnalySIS (Soft Imaging System GmbH, Germany) or ImageJ Fiji software. The numerical data obtained were processed using Microsoft Excel.

To determine statistical significance, Student's *t*‐test and ANOVA tests were conducted either using Excel, GraphPad Prism (GraphPad Software Inc.) or on‐line calculators (http://in-silico.net, and http://statpages.info/anova1sm.html). *p*‐values of less than 0.05 indicated a significant difference among the various groups. For multiple‐comparison experiments, the Kruskal–Wallis or Tukey Honestly Significant Difference post hoc test was used.

## CONFLICT OF INTEREST STATEMENT

The authors declare no conflicts of interest.

## Supporting information

Supporting information.

Supporting information.

Supporting information.

Supporting information.

Supporting information.

Supporting information.

Supporting information.

Supporting information.

Supporting information.

Supporting information.

Supporting information.
