# Supplementary figures and images for: Chitosan stimulates root hair callose deposition, endomembrane dynamics, and inhibits root hair growth
Source: Plant Cell Environ. 2024 Sep 13;48(1):451–69. doi: 10.1111/pce.15111 (PMC11615431; doi:10.1111/pce.15111)

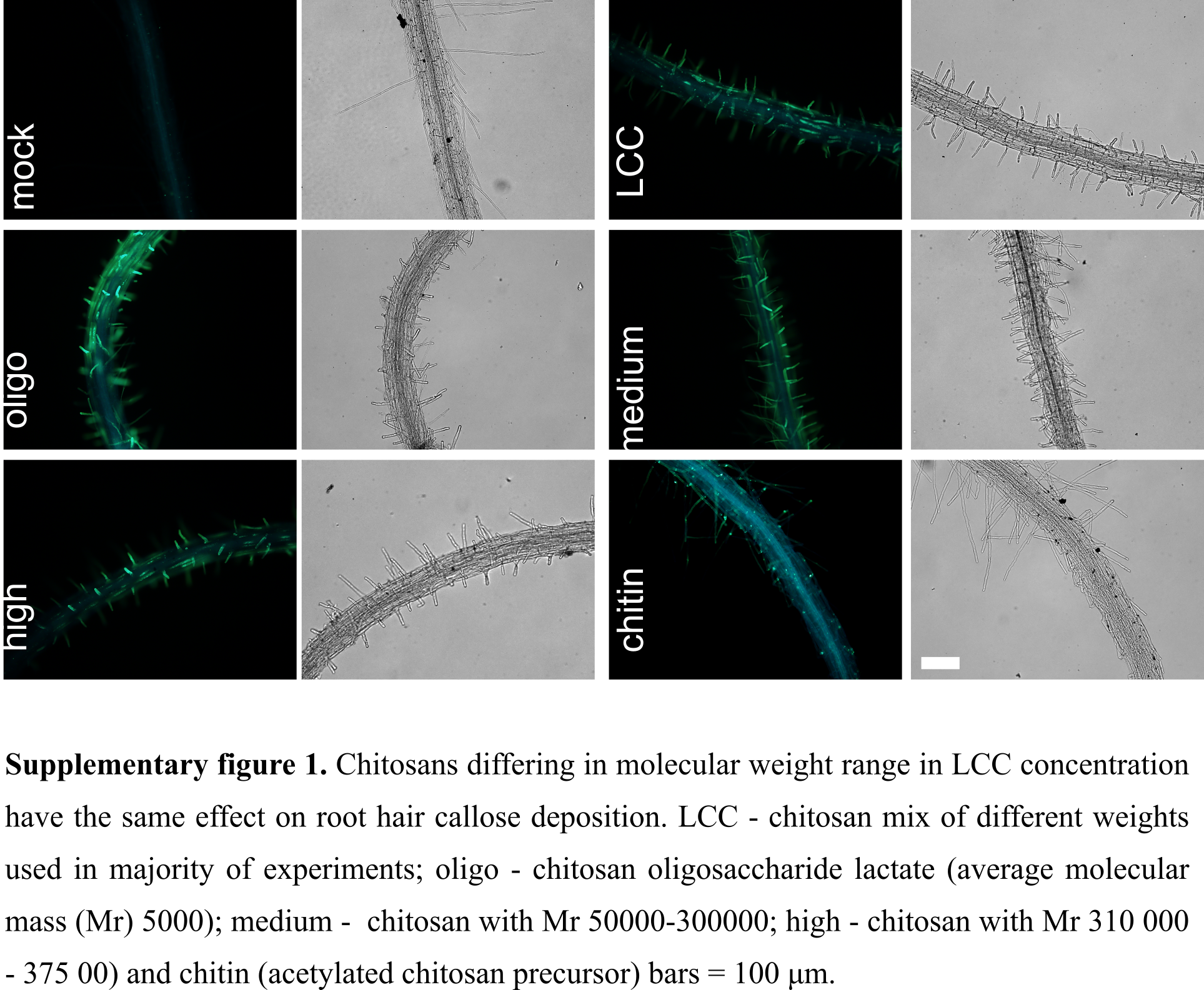

Supplement: Supplementary file 1 — Supporting information. [file PCE-48-451-s008.tiff]

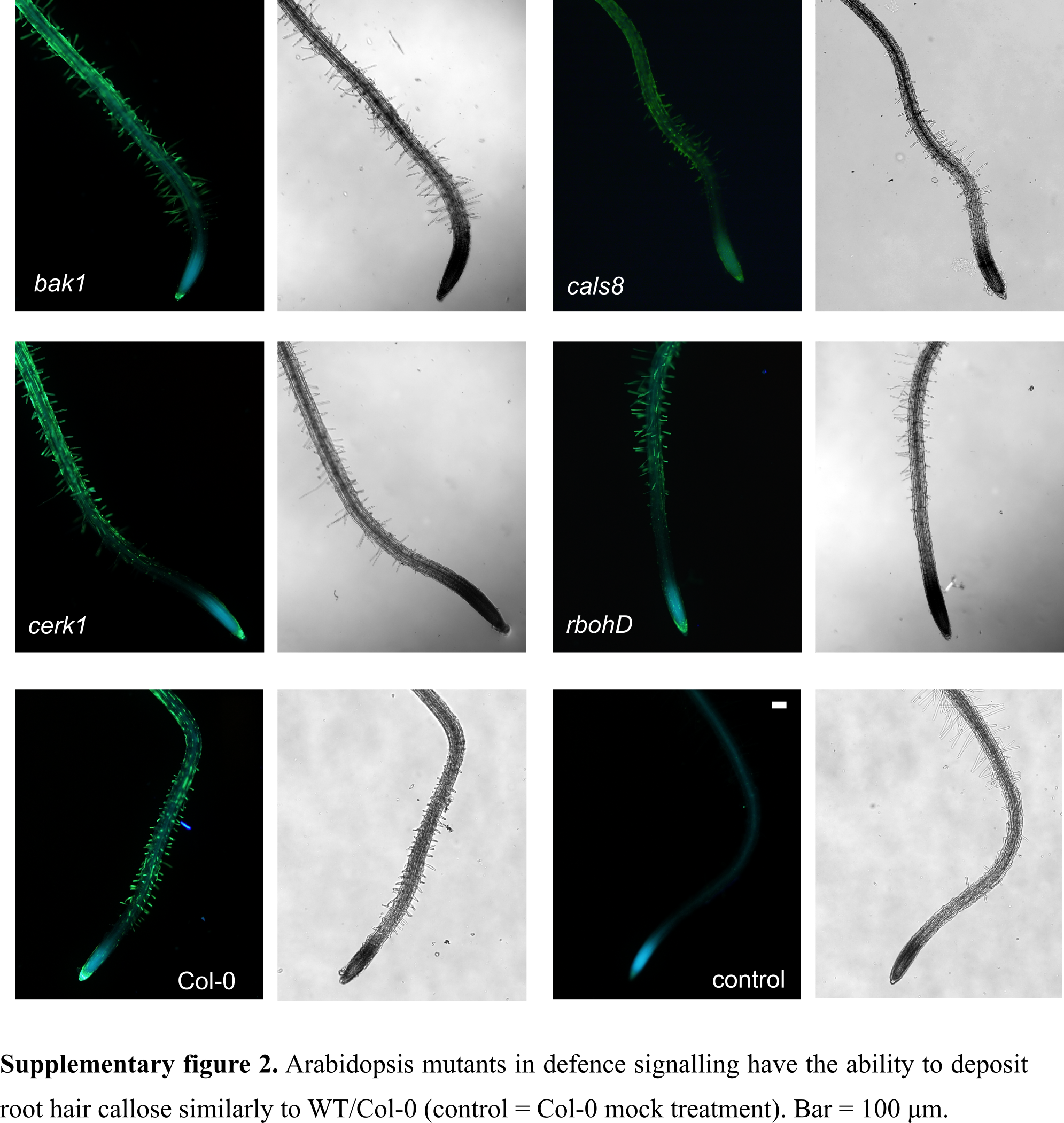

Supplement: Supplementary file 2 — Supporting information. [file PCE-48-451-s007.tiff]

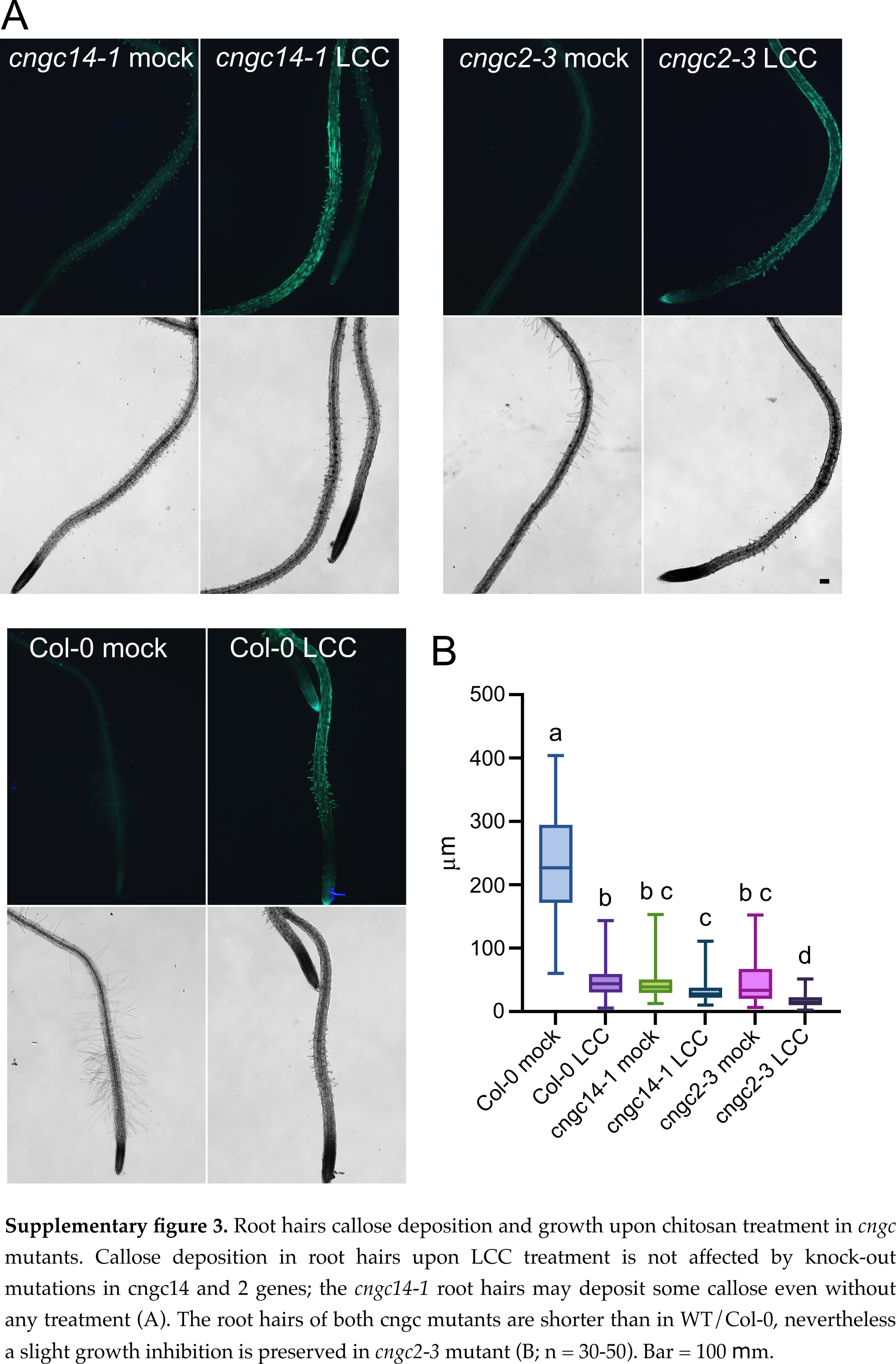

Supplement: Supplementary file 3 — Supporting information. [file PCE-48-451-s004.tiff]

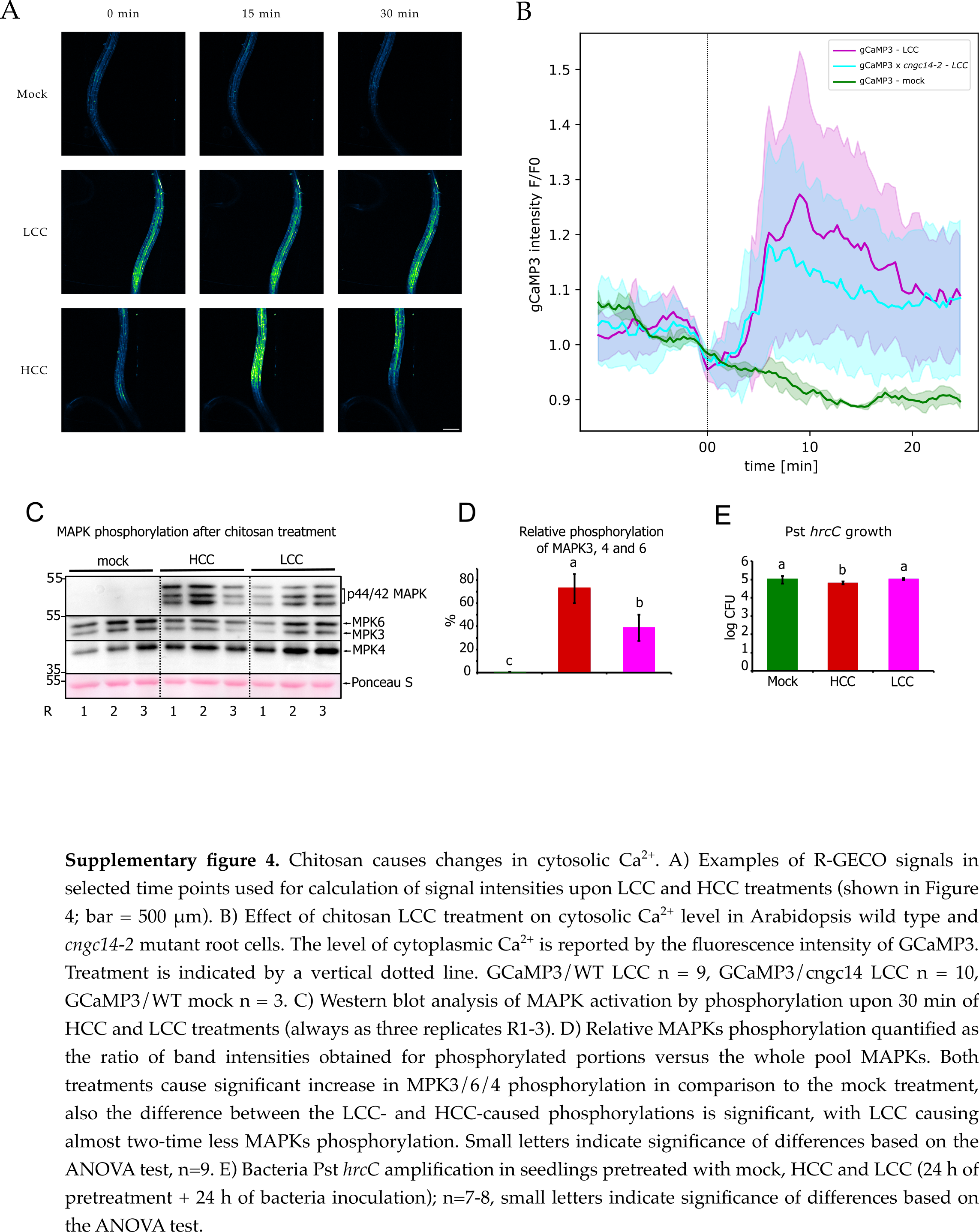

Supplement: Supplementary file 4 — Supporting information. [file PCE-48-451-s009.tiff]

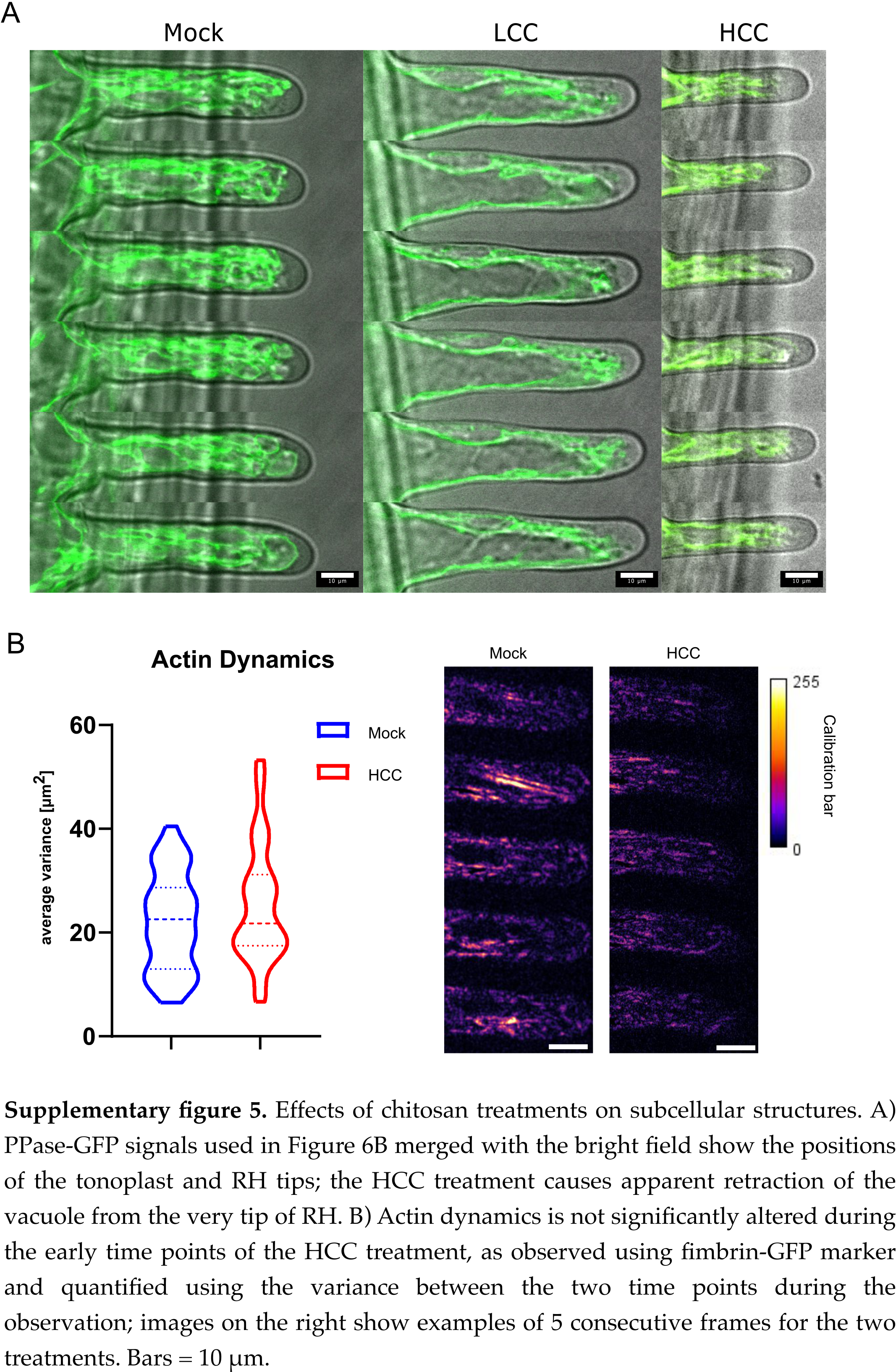

Supplement: Supplementary file 5 — Supporting information. [file PCE-48-451-s006.tiff]

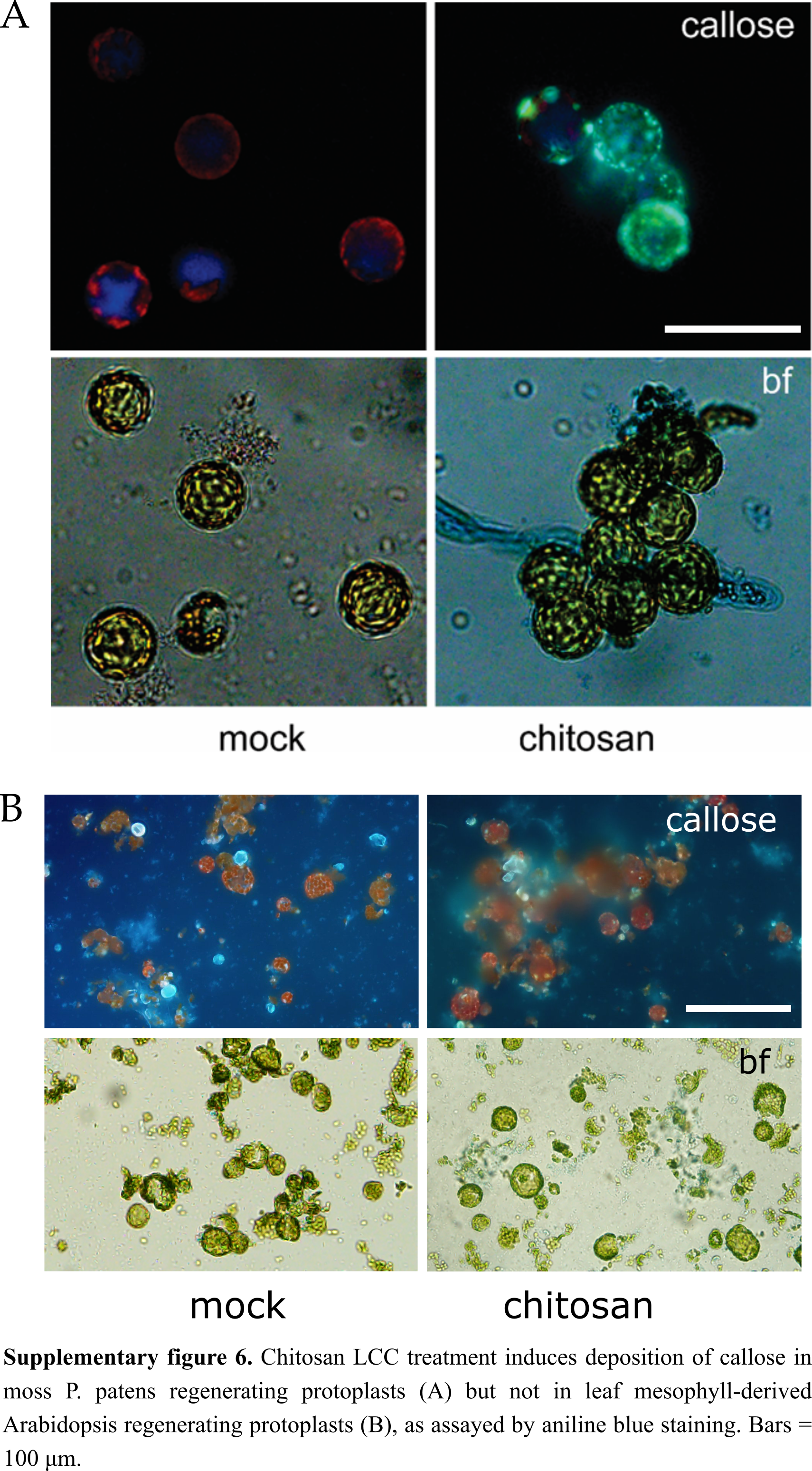

Supplement: Supplementary file 6 — Supporting information. [file PCE-48-451-s011.tiff]
