# Supplementary material for: Chitosan stimulates root hair callose deposition, endomembrane dynamics, and inhibits root hair growth
Source: Plant Cell Environ. 2024 Sep 13;48(1):451–69. doi: 10.1111/pce.15111 (PMC11615431; doi:10.1111/pce.15111)
Supplement: Supplementary file 8 — Supporting information. [file PCE-48-451-s010.docx]

| **HCC (seedlings)** |  |  |  |
| --- | --- | --- | --- |
| **gene_ID** | **symbols** | **description** | **fc log** |
| *DOWN* |  |  |  |
| AT5G04960 |  | Plant invertase/pectin methylesterase inhibitor | -5,39 |
| AT3G26610 |  | Pectin lyase-like superfamily protein | -4,19 |
| AT1G65570 |  | Pectin lyase-like superfamily protein | -3,64 |
| AT5G14650 |  | Pectin lyase-like superfamily protein | -3,09 |
| AT1G57590 |  | Pectinacetylesterase family protein | -2,90 |
| AT4G25250 |  | Plant invertase/pectin methylesterase inhibitor | -2,89 |
| AT5G04970 |  | Plant invertase/pectin methylesterase inhibitor | -2,86 |
| AT1G02810 |  | Plant invertase/pectin methylesterase inhibitor | -2,80 |
| AT1G05650 |  | Pectin lyase-like superfamily protein | -2,62 |
| AT1G53830 | PME2 | pectin methylesterase 2 | -2,53 |
| AT5G62340 |  | Plant invertase/pectin methylesterase inhibitor | -2,53 |
| AT5G51490 |  | Plant invertase/pectin methylesterase inhibitor | -2,47 |
| AT3G59850 |  | Pectin lyase-like superfamily protein | -2,39 |
| AT4G00080 | UNE11 | Plant invertase/pectin methylesterase inhibitor | -2,19 |
| AT1G70720 |  | Plant invertase/pectin methylesterase inhibitor | -2,07 |
| AT2G43880 |  | Pectin lyase-like superfamily protein | -1,87 |
| AT5G47500 |  | Pectin lyase-like superfamily protein | -1,84 |
| AT5G46960 |  | Plant invertase/pectin methylesterase inhibitor | -1,66 |
| AT1G05660 |  | Pectin lyase-like superfamily protein | -1,63 |
| AT1G60590 |  | Pectin lyase-like superfamily protein | -1,43 |
| AT3G24670 |  | Pectin lyase-like superfamily protein | -1,20 |
| AT3G53190 |  | Pectin lyase-like superfamily protein | -1,12 |
| AT1G10640 |  | Pectin lyase-like superfamily protein | -1,11 |
| AT5G19730 |  | Pectin lyase-like superfamily protein | -1,11 |
| *UP* |  |  |  |
| AT3G09405 |  | Pectinacetylesterase family protein | 2,98 |
| AT1G14890 |  | Plant invertase/pectin methylesterase inhibitor | 1,61 |
| AT2G43870 |  | Pectin lyase-like superfamily protein | 1,19 |
|  |  |  |  |
|  |  |  |  |
|  |  |  |  |
