# Supplementary material for: Chitosan stimulates root hair callose deposition, endomembrane dynamics, and inhibits root hair growth
Source: Plant Cell Environ. 2024 Sep 13;48(1):451–69. doi: 10.1111/pce.15111 (PMC11615431; doi:10.1111/pce.15111)
Supplement: Supplementary file 9 — Supporting information. [file PCE-48-451-s003.docx]

| **Name** | **Sequence** |
| --- | --- |
| AtACS6_qFor1 | AGCCAACTTGAGGAGGAGAC |
| AtACS6_qRev1 | CGAATGAGGCGAGAAGAAGC |
| AtCML37_qFor1 | GGTGGAGGAAGTGGTGAAGA |
| AtCML37_qRev1 | CGCCGCCGTAATAAACTCTT |
| AtDisResPrxy_qFor1 | GCAGGGAATTGGACAGCTTT |
| AtDisResPrxy_qRev1 | CTCCCGAAGCCAACTCCTAA |
| AtDRP1A_qFor1 | CAGCTGGTTGACATGGAGTG |
| AtDRP1A_qRev1 | CGAAAATGGAGTGTGTGGGG |
| AtERF11_qFor1 | GCACCGTGGAATCATCGTTT |
| AtERF11_qRev1 | AACACCATCATCCCAGCCTT |
| AtLIP1_qFor1 | AGCAGGGGTTGACGTTATGA |
| AtLIP1_qRev1 | TTTGTACGACGACCTCACCA |
| AtLRRxy_qFor1 | CGTTGCCGGAGACTATAGGT |
| AtLRRxy_qRev1 | TGAAGTTCTCGAGCCTTGGT |
| AtUGD2_qFor1 | CGATGTGTGCAAGGGTCTTT |
| AtUGD2_qRev1 | ACTGTTGTTGGGCTCATTGG |
| AtWRKY46_qFor1 | GAAACCGACCAAGTCCGAAG |
| AtWRKY46_qRev1 | CCTCGCTGGTAATTGCAGAC |
