# Supplementary material for: Chitosan stimulates root hair callose deposition, endomembrane dynamics, and inhibits root hair growth
Source: Plant Cell Environ. 2024 Sep 13;48(1):451–69. doi: 10.1111/pce.15111 (PMC11615431; doi:10.1111/pce.15111)
Supplement: Supplementary file 11 — Supporting information. [file PCE-48-451-s002.docx]

**Supplementary figure 1.**  Chitosans differing in molecular weight range in LCC concentration have the same effect on root hair callose deposition. LCC - chitosan mix of different weights used in majority of experiments; oligo - chitosan oligosaccharide lactate (average molecular mass (Mr) 5000); medium - chitosan with Mr 50000-300000; high - chitosan with Mr 310000 - 375000) and chitin (acetylated chitosan precursor) Bar=100 μm.

**Supplementary figure 2.** Arabidopsis mutants in defence signalling have the ability to deposit root hair callose similarly to WT/Col-0 (control = Col-0 mock treatment) . Bar = 100 μm.

**Supplementary figure 3.** Root hairs callose deposition and growth upon chitosan treatment in *cngc* mutants. Callose deposition in root hairs upon LCC treatment is not affected by knock-out mutations in cngc14 and 2 genes; the *cngc14-1* root hairs may deposit some callose even without any treatment (A). The root hairs of both *cngc* mutants are shorter than in WT/Col-0, nevertheless a slight growth inhibition is preserved in *cngc2-3* mutant (B; n = 30-50). Bar = 100 μm.

**Supplementary figure 4**. Chitosan causes changes in cytosolic Ca^2+^. A) Examples of R-GECO signals in selected time points used for calculation of signal intensities upon LCC and HCC treatments (shown in Figure 4; bar = 500 μm). B) Effect of chitosan LCC treatment on cytosolic Ca^2+^ level in Arabidopsis wild type and *cngc14-2* mutant root cells. The level of cytoplasmic Ca^2+^ is reported by the fluorescence intensity of GCaMP3. Treatment is indicated by a vertical dotted line. GCaMP3/WT LCC n = 9, GCaMP3/*cngc14* LCC n = 10, GCaMP3/WT mock n = 3. C) Western blot analysis of MAPK activation by phosphorylation upon 30 min of HCC and LCC treatments (always as three replicates R1-3). D) Relative MAPKs phosphorylation quantified as the ratio of band intensities obtained for phosphorylated portions versus the whole pool MAPKs. Both treatments cause significant increase in MPK3/6/4 phosphorylation in comparison to the mock treatment, also the difference between the LCC- and HCC-caused phosphorylations is significant, with LCC causing almost two-time less MAPKs phosphorylation. Small letters indicate significance of differences based on the ANOVA test, n=9. E) Bacteria Pst *hrcC* amplification in seedlings pretreated with mock, HCC and LCC (24 h of pretreatment + 24 h of bacteria inoculation); n=7-8, small letters indicate significance of differences based on the ANOVA test.

**Supplementary figure 5.** Effects of chitosan treatments on subcellular structures. A) PPase-GFP signals used in Figure 6B merged with the bright field show the positions of the tonoplast and RH tips; the HCC treatment causes apparent retraction of the vacuole from the very tip of RH. B) Actin dynamics is not significantly altered during the early time points of the HCC treatment, as observed using fimbrin-GFP marker and quantified using the variance between the two time points during the observation; images on the right show examples of 5 consecutive frames for the two treatments.

**Supplementary figure 6.** Chitosan LCC treatment induces deposition of callose in moss *P. patens* regenerating protoplasts (A) but not in leaf mesophyll-derived Arabidopsis regenerating protoplasts (B), as assayed by aniline blue staining. Bars = 100 μm.

**Supplementary video 1.** R-GECO1 signal intensity changes in root hairs/root in response to mock and subsequent HCC treatment (false coloured in Green Fire Blue LUT). Time legend is located on the upper left corner, scale bar 100 μm.

**Supplementary Table S1.** RNAseq data with lists of differentially expressed genes (DEGs) and GO enrichment categories for chitosan treatments (LCC and HCC).

**Supplementary Table S2.** List of DEGs with functions related to pectin modification found in RNAseq analysis of chitosan treated seedlings.

**Supplementary Table S3.** List of primers used for Q-RT-PCR.
